# Knockout of *mafba* Causes Inner-Ear Developmental Defects in Zebrafish via the Impairment of Proliferation and Differentiation of Ionocyte Progenitor Cells

**DOI:** 10.3390/biomedicines9111699

**Published:** 2021-11-16

**Authors:** Xiang Chen, Yuwen Huang, Pan Gao, Yuexia Lv, Danna Jia, Kui Sun, Yunqiao Han, Hualei Hu, Zhaohui Tang, Xiang Ren, Mugen Liu

**Affiliations:** Key Laboratory of Molecular Biophysics of Ministry of Education, College of Life Science and Technology, Huazhong University of Science and Technology, 1037 Luoyu Road, Wuhan 430074, China; xiangchenhust816@outlook.com (X.C.); yuwenhuang_1994@foxmail.com (Y.H.); gaopan924989055@163.com (P.G.); lyuexia0614@163.com (Y.L.); jiadanna@foxmail.com (D.J.); shymcg@163.com (K.S.); 18064097079@163.com (Y.H.); d202080697@hust.edu.cn (H.H.); zh_tang@hust.edu.cn (Z.T.)

**Keywords:** zebrafish, *mafba*, cell proliferation, cell differentiation, ion homeostasis, inner-ear development

## Abstract

Zebrafish is an excellent model for exploring the development of the inner ear. Its inner ear has similar functions to that of humans, specifically in the maintenance of hearing and balance. Mafba is a component of the Maf transcription factor family. It participates in multiple biological processes, but its role in inner-ear development remains poorly understood. In this study, we constructed a *mafba* knockout (*mafba^−/−^*) zebrafish model using CRISPR/Cas9 technology. The *mafba^−/−^* mutant inner ear displayed severe impairments, such as enlarged otocysts, smaller or absent otoliths, and insensitivity to sound stimulation. The proliferation of p63^+^ epidermal stem cells and dlc^+^ ionocyte progenitors was inhibited in *mafba^−/−^* mutants. Moreover, the results showed that *mafba* deletion induces the apoptosis of differentiated K^+^-ATPase-rich (NR) cells and H^+^-ATPase-rich (HR) cells. The activation of p53 apoptosis and G0/G1 cell cycle arrest resulted from DNA damage in the inner-ear region, providing a mechanism to account for the inner ear deficiencies. The loss of homeostasis resulting from disorders of ionocyte progenitors resulted in structural defects in the inner ear and, consequently, loss of hearing. In conclusion, the present study elucidated the function of ionic channel homeostasis and inner-ear development using a zebrafish Mafba model and clarified the possible physiological roles.

## 1. Introduction

Approximately 360 million people are born with congenital and progressive hearing defects worldwide [1]. Genetic defects account for approximately 50% of hearing loss [2], most of which is caused by mutations in genes associated with inner-ear development [3]. The vertebrate inner ear is a conserved sensory organ responsible for vestibular and auditory functions [4]. It plays an important role in detecting body acceleration and keeping balance [5,6]. The mammalian ear is composed of inner, middle, and external components, while the zebrafish, belonging to the teleost fish, only exhibits the inner ear. Although there is considerable structural diversity of the inner ears among different species, the molecular basis of their development is relatively conserved [7]. Zebrafish has become an excellent vertebrate model for exploring the mechanism of inner-ear development and related diseases in recent years [8,9]. There is an urgent need to enhance our understanding of the molecular mechanisms of inner-ear development and to pragmatize biological strategies for restoring auditory functions.

The zebrafish otic vesicles consist of sensory hair cells and other non-sensory epithelial cells [10]. The barrier functions of the inner ear epithelial cells are essential to maintain homoeostasis in the otic lumen and the endolymph, which guides the development of hair cells, semicircular canals, and otoliths [11,12]. Ions and other components are secreted into the otic lumen and endolymph through ion channels of otic epithelial cells’ membranes to maintain the steady state of endolymph homeostasis [13], which is also crucial for otolith formation [8,9,14,15]. Both the otic epithelium and the inner-ear epidermis regulate osmotic homeostasis through transporting ions and acid-base equivalents. In zebrafish embryos, the p63^+^ epidermal stem cells become ionocytes or keratinocytes, determined by DeltaC (dlc)-Notch-mediated lateral inhibition [16,17]. The differentiated ionocytes develop mainly in the skin of embryos and maintain body fluid ionic homeostasis. Researchers have identified five types of ionocytes in zebrafish embryos, including NR cells, HR cells, SLC26-expressing cells, Na^+^-Cl^−^cotransporter-expressing (NCC) cells, and K^+^-secreting (KS) cells [18]. These cells regulate osmotic homeostasis by utilizing various ion transporters [19,20]. Intriguingly, the specification and differentiation of ionocytes is controlled by a positive feedback loop between *foxi3a* and *foxi3b* [17,21,22]. It activates some important downstream transcription factors, such as *glial cell missing* 2 (*gcm2*), to determine which ionocyte progenitors differentiate into HR or NR cells following the upregulated expression of *foxi3a* or *foxi3b*, respectively [23]. The down-regulated expression of Na^+^-K^+^-Cl^−^ cotransporter *nkcc1* (*slc12a2*) and a few Na^+^/K^+^-ATPase channel genes results in a collapse of the otic vesicle in zebrafish embryos and the loss of endolymphatic fluid [24,25]. Consequently, the distribution of ion channels in the otic epithelial cells and inner-ear epidermis is essential for the homeostasis maintenance and inner-ear development.

Members of the MAF family are divided into two subfamilies. Large Maf family members (Mafa, Mafb, c-Maf, and Nrl) contain a C-terminal basic leucine-zipper domain (b-Zip), which mediates dimerization and DNA binding, and an N-terminal transactivation domain (TAD), which regulates target gene transcription. A small Maf family (Mafk, Mafg, and Maff) contains only a b-Zip domain [26]. The transcription factor *mafb* plays crucial roles in epidermal keratinocyte differentiation in the mammalian epidermis [27]. Numerous studies have identified the role of Mafb in the developmental of various tissues and in cellular differentiation. However, there is, as yet, no report on the association between ionocytes and *mafb*. For instance, *mafb* deprivation impairs the differentiation of podocyte, osteoclast, monocyte, and epidermal cells [27,28,29,30], the development of pancreatic islet α/β-cells [31], and the parathyroid gland [32] in mammals. Losses or decreases in the expression of mouse *mafb* may cause atopic dermatitis (AD) and psoriasis vulgaris [27]. In addition, *mafb* mutations in humans and mice lead to multicentric carpotarsal osteolysis (MCTO) [33], Duane retraction syndrome (DRS), aberrant extraocular muscle innervation, and inner-ear defects [34]. Prior studies have shown that *kreisler*/*mafb* mutant mice display inner-ear defects, which may be affected by abnormal adjacent segmented hindbrain development and signal transduction [35]. Silencing of the *valentino*/*mafba* in zebrafish impairs the signal regulation of *fgf3* in the hindbrain, which leads to the abnormal development of the hindbrain and otic vesicle [36]. In addition, a previous study showed that *mafb* deficiency promotes tumorigenesis in clinical colorectal cancer (CRC) and causes cell cycle arrest in the G0/G1 phase [37]. The balance of *mafb* and *cFos* or *cJun* heterodimer complexes controls the apoptosis-survival fate, which is required for triggering p53-dependent apoptosis in response to DNA damage [38]. These studies reveal the role of Mafb in regulating cell survival and various developmental processes. However, the specific mechanism of how *mafb* mutants cause inner-ear defects is poorly understood. Here, we suggest that the proliferation and differentiation of ionocytes are directly influenced by the transcription factor *mafba*, and then ion homeostasis, which affects the inner ear.

We constructed a zebrafish *mafba* (the homologous gene of mammalian *mafb*) knockout model using CRISPR/Cas9 technology. Loss of Mafba impairs inner-ear development in zebrafish embryos. The inner-ear defect in *mafba^−/−^* embryos results from a loss of ionic homeostasis, due to reduced populations of ionocyte progenitors and the apoptosis of HR and NR ionocytes in the otic epithelium. We further confirmed that *mafba* deletion induces apoptosis in HR and NR cells, resulting from G0/G1 cell cycle arrest, which is associated with DNA damage. Our findings provide a novel insight into Mafba’s role in the maintenance of ionic channel homeostasis and inner-ear development.

## 2. Materials and Methods

### 2.1. Zebrafish Maintenance

Zebrafish (*Danio rerio*) larvae and adults were raised in 28.5 °C-constant temperature recirculating water under a 14-h light/10-h dark cycle [39]. We collected and maintained embryos in E3 medium for 72 h until the larvae hatched. If needed, we added 0.003% phenylthiourea to the E3 medium at 12 h after fertilization to inhibit pigmentation of embryos. We anaesthetized the embryos and larvae with 0.02% tricaine before further analysis. The specific developmental stages of zebrafish are defined as hours postfertilization (hpf) and days postfertilization (dpf). All zebrafish experimental procedures were performed in accordance with guidelines approved by the Experimental Animal Ethics Committee of Huazhong University of Science and Technology.

### 2.2. Generation of Mafba Mutant by CRISPR/Cas9 Technology

The target sites were designed using a web tool (http://chopchop.cbu.uib.no/, accessed on 30 August 2019). The target sequences of *mafba* we used are listed in Table S1. The gRNA and Cas9 mRNA were synthesized with a TranscriptAid T7 High Yield Transcription Kit (Thermo Scientific, Waltham, MA, USA) and mMESSAGE mMACHINE T7 transcription synthesis kit (Invitrogen, Carlsbad, CA, USA), respectively. A mixture of 600 pg Cas9 mRNA and 200 pg gRNA were co-injected into the one-cell-stage, wild-type embryos. At 3 dpf, 18 injected zebrafish embryos were collected to identify F0 founders by genotyping in order to evaluate CRISPR/Cas9 system target efficiency. A 464-bases pair (bp) DNA product containing the *mafba* targeting site was amplified by PCR (primers used are listed in Table S1) and sequenced. The F1 generation was obtained through the outcrossing of F0 mutant zebrafish to wild-type zebrafish. The F2 generation of mutant zebrafish were obtained and raised to adults using the same method as for the F1 generation described above. The F2 generation *mafba^+/−^* zebrafish were crossed to produce the sibling (*mafba^+/+^*), *mafba^+/−^,* and *mafba^−/−^* mutant embryos.

### 2.3. Whole-Mount In Situ Hybridization

Whole-mount in situ hybridization (WISH) for zebrafish embryos was executed as previously described [40]. The sequences of all probes and primers are listed in Table S1. The images were acquired using an optical microscope (10 × lens, BX53, Olympus, Tokyo, Japan). After imaging, we identified the genotypes by extracting DNA from the embryos. The numbers at the bottom right of each picture indicate the number of embryos with a similar staining pattern across all embryos examined.

### 2.4. Whole-Mount Immunofluorescence Assay

Whole-mount immunofluorescence was performed as described [41,42]. The embryos were fixed with 4% paraformaldehyde at 4 °C overnight. After three washes with 0.5% PBST (PBS + 0.5% Triton X-100) for 10 min each, embryos were permeabilized in solution (PBS + 2% Triton X-100) to dissolve the otolith at room temperature for 8 h (time until the otolith is completely dissolved may be longer). Then, we washed the embryos with 0.5% PBST twice and blocked with them 10% goat serum in 0.5% PBST for 2 h. Next, the embryos were incubated with primary antibodies (diluted in 10% goat serum in 0.5% PBST) overnight at 4 °C. The following primary antibodies were used: p63 (Ab735, Abcam; 1:50, Cambridge, UK), phosphorylated histone H3 (AF3358, Affinity; 1:200), p63 (Ab124762, Abcam; 1:100), Dlc (Ab7336, Abcam; 1:100), Atp1a1a.4 (p09572, DHSB; 1:50), Atp6v1a (GTX129736, GeneTex; 1:100), γH2AX (9178s, CST; 1:200), Myo7a (20720, Proteintech; 1:200), Acetylated-α-Tubulin (sc-23950, Santa Cruz Biotechnology; 1:100), Mafba (GTX128295, GenxTex; 1:100), and Alexa Fluor 594 Phalloidin (A12381, Thermo Scientific; 1:200). After 0.5% PBST washes, the embryos were treated with the corresponding secondary antibody (Alexa-Fluor 405-nm, Alexa-Fluor 488-nm, or 594-nm secondary antibody, 1:500, diluted in 10% goat serum of 0.5% PBST) at room temperature in the dark for 3 h. For the double immunofluorescence labeling, the samples continued treatment in accordance with the standard immunofluorescence procedure described above. We washed the embryos with PBST twice and stored them in PBS at 4 °C. The samples images were captured using a confocal microscope (FV31S-SW, Olympus) with a 10 × lens (NA = 0.40), fully automatic and continuously adjustable in the range of 50–800 nm, independent spectral fluorescence detector, and fluorescence detection channel. The spectral windows were blue (359–461 nm), green (500–580 nm), and red (610–700 nm), respectively. Histogram normalizations and gamma adjustments were adjusted before the image acquisition, and we did not make adjustments at the later stage. Image stitching and projections were carried out through the ImageJ v1.8.0 software [43,44].

### 2.5. EDU and TUNEL Assay

Zebrafish embryos were treated with 2 mM EDU (5-ethynyl-2′- deoxyuridine) for 30 min at 4 °C in egg-water. The embryos were transferred to fresh E3 medium for 2 h and fixed in 4% paraformaldehyde at 4 °C overnight. We treated the zebrafish embryos with Cell-Light™ EdU Cell Proliferation Detection (C10310-1, Ribobio, Guangzhou, China), according to the operating protocol, to visualize the proliferating cells. For the triple labeling of EDU, p63, and dlc, the samples were continuously treated in accordance with the standard immunofluorescence procedure and assessed via a confocal microscope (FV31S-SW, Olympus), as mentioned in Section 2.4.

TUNEL staining was performed as previously described [45]. The apoptosis cells were labeled with the TUNEL BrightRed Apoptosis Detection Kit (Vazyme Biotech, NanJing, China). For double labeling of the TUNEL and atp1a1a.4 and the TUNEL and atp6v1a, as well as triple labeling the TUNEL, p63, and dlc, the samples were treated in accordance with the standard immunofluorescence procedure, and assessed by a confocal microscope (FV31S-SW, Olympus), as mentioned in Section 2.4.

### 2.6. Western Blot

Zebrafish embryos were identified and collected for protein extraction at 4 dpf. Approximately 25 heads of the same genotypes, including the inner-ear region, were lysed with RIPA lysis buffer containing cOmplete protease inhibitor cocktail (04693132001, Roche, Basel, Switzerland), while the tails of embryos were used for the genotyping assay. Western blot was performed as described previously [46]. The following primary antibodies were used in this study: anti-Mafb (PA5-40756, Thermo Scientific; 1:500), anti-p53 (3121, Daian; 1:1000), anti-γH2AX (9178s, CST; 1:1000), anti-GAPDH (60004, Proteintech; 1:1000), anti-CDK2 (R1309, HUABIO; 1:1000), anti-CDK6 (ER40101, HUABIO; 1:1000), anti-Cyclin D1 (0427, HUABIO; 1:1000), anti-Cyclin E1 (ER1906, HUABIO; 1:1000), anti-PCNA (10205, Proteintech; 1:2000), anti-Phospho-ATR (2853, CST; 1:1000), anti-Phospho-ATM (13050, CST; 1:1000), and anti-Rb1 (10048, Proteintech; 1:1000). The images were captured with a ChemiDocTM XRS^+^ system (BIO-RAD).

### 2.7. Bioinformatics

All relevant Maf factor structures and sequences are available at Genome Browser (http://genome.ucsc.edu/cgi-bin/hgBlat, accessed on 12 July 2020) and Ensembl (http://asia.ensembl.org/, accessed on 12 July 2020). SWISS-MODEL (https://swissmodel.expasy.org/, accessed on 12 July 2020) provided the fully automated protein structure homology modeling [47]. The amino acid sequences of Maf family factors from the other species mentioned above were downloaded from the NCBI GenBank database (http://www.ncbi.nlm.nih.gov/protein, accessed on 12 July 2020) and used for the multiple sequence alignments performed by ClustalW software.

### 2.8. Comet Assay

Heads from 30 zebrafish embryos were cut off and placed in 1.5 mL of ice-cold PBS (PBS + 20 mM EDTA). The cell suspensions were prepared by mincing the tissues. Alkaline Comet Assay was carried out in accordance with the manufacturer’s procedure using the Comet Assay Kit (Abcam, Ab238544) [48]. DNA damage in single-strand breaks (SSB) and double-strand breaks (DSB) can be detected by alkaline electrophoresis. Images were captured by fluorescence microscopy (Nikon Ti_i80).

### 2.9. Startle Response Tests

The sound-evoked C-start escape response was examined in 96-well plastic plates and recorded with a high-speed camera (Olympus, E-M10, 1000 fps) under infrared light illumination. Pure tone stimulations (10 ms, 500 Hz) with two different intensities (20 dB and 40 dB) were supplied through a plastic board mounted on a voice box (HiVi, D1080MKII). Positive C-startle response was confirmed if the response occurred within 20 ms after the application of stimuli. For each group, 32 larvae were tested. Each larva was tested 10 times and each larva’s C-startle times was computed. The possibility of measuring a C-startle response in a group of larvae was defined as the average percentage of C-startle [49].

### 2.10. Quantitative RT-PCR Analysis

Zebrafish embryos at 4 dpf were collected for total RNA extraction. The tails were dissected and used for genotyping. The residual head parts of approximately 30 were cut off and blended together to extract RNA by using the TRIzol Reagent (Vazyme Biotech, NanJing, China). They were then reverse transcribed into cDNA using HiScript^®^ II Q RT SuperMix (+gDNA wiper) for qPCR (Vazyme Biotech, Nanjing, China). The qPCR was carried out with AceQ^TM^ qPCR SYBR Green Master Mix (Vazyme Biotech, Nanjing, China) in a StepOnePlus™ real time PCR machine, and analyzed with Graphpad 6.1 software. The relative mRNA expression levels of the selected genes were calculated using the 2^−^^△△Ct^ method [50]. The β-actin was set as an internal control. All qPCR primers used in this research are shown in Supplementary Table S1.

### 2.11. Flow Cytometry Analysis for Cell Cycle

For 4-dpf fish, 100 embryos were collected and washed once in ice-cold PBS. We removed all the solution into a 50-mL Falcon tube, then passed it through a 100-µm nylon filter (BD Falcon, Cat. 352360, Franklin Lakes, NJ, USA). We aspirated the solution and resuspended the pellet in 1 mL of Dispase II (Gibco, 1U/mL in PBS, Shanghai, China) to break up tissues. The solution was transferred into a 1.5-mL tube and incubated at 37 °C for 20 min. Then 1 mL of washing buffer (Hanks buffered saline solution containing 20% FBS, 5 mM CaCl_2_, and 0.5 u/mL DNAse) was added to the samples. The samples were treated with 0.2 mL of PBT (100 µg/mL RNase A and 50 00B5g/mL PI in PBS). Setting 200 µL PBS as the control group, we commenced dark treatment at room temperature for 30 min. We resuspended the cells in 1 mL PBS (with 2–3% FBS), which was then passed through a 40-µm nylon filter (BD Falcon, Cat. 352340, Franklin Lakes, NJ, USA), and then we counted the cells. The labeled cells were detected for PI staining using a Beckman Coulter CytoFlex 5, and the percentages of cells in the G0/G1, S, and G2/M phases in each sample were determined via gating using the Flowjo 10.0 software (Beckman Coulter, Brea, CA, USA).

### 2.12. Statistical Analysis

Statistical analyses were performed using GraphPad Prism 6.0 software. The experiments were repeated at least three times. The numbers of samples measure in every experiment are discussed in the Section 2 and in the figure legends. The two-tailed Student’s *t*-test was used for determining significance. The results are presented as the mean ± SD. The level of significance was set to *p* < 0.05, and *p* > 0.05, *p* < 0.05, *p* < 0.01, *p* < 0.001, and *p* < 0.0001 were labeled as ns, *, **, ***, and ****, respectively.

## 3. Results

### 3.1. Generation of mafba^−/−^ Mutant Zebrafish with CRISPR/Cas9 Technology

There are two *mafba* transcripts (*mafba*-201(NM_131015.3) and *mafba*-202 (AB006322.1)) listed in the ZFIN database. Each contains only one exon, encoding 397 and 356 amino acids, respectively. Both can be detected in zebrafish embryos. Zebrafish Mafba proteins are 73%/72% identical at the amino acid level to the human/mouse Mafb protein, respectively. These are significantly higher than the sequence similarities among zebrafish Mafba, Mafbb, and Mafa proteins (Figure S1A). The functional domains of the Maf family members are highly conserved among zebrafish and mammalian Mafb proteins, including N-terminal transcriptional activation domain (TAD), the C-terminal basic domain (BR), and the leucine zipper domain (LZ) (Figure S1A,C), which are the functional domains of Maf family members [26]. All of the tertiary structures consist of three α-helixes and two β-sheets sharing many similarities (Figure S1B). In vertebrates, small Maf evolves into three members (MafF, MafG, and MafK), while large Maf evolves into four members (MafA, MafB, C-MAF, and Nrl). Some of the genes duplicate because of an extra copy of the genome in the teleost [51]. Zebrafish have five large Maf genes and four small Maf genes. Due to an extra copy of the genome in the teleost fish, gene duplication may occur in both MafB and MafG [52].

**Figure 1:**
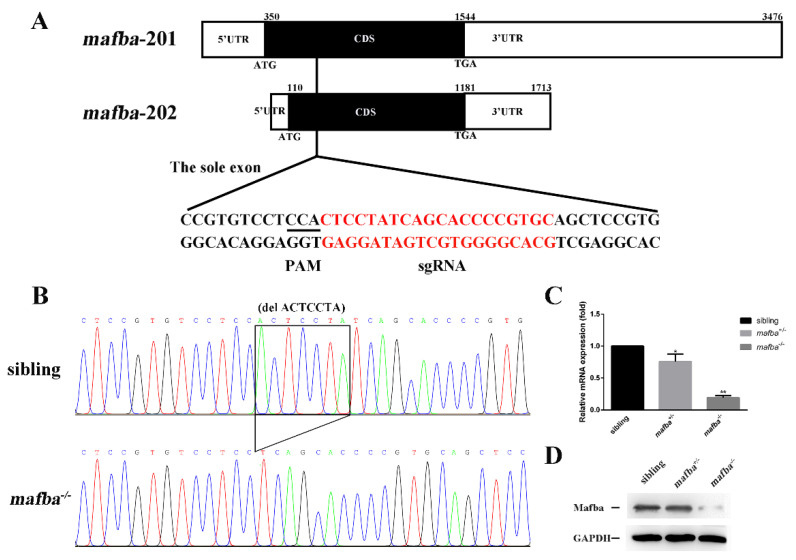
CRISPR/Cas9-mediated mutagenesis of *mafba.* (**A**) The sole exon of two *mafba* transcripts and the CRISPR/Cas9 target site are shown. The 5′UTR, CDS, and 3′UTR regions of the two *mafba* transcripts are shown in detail. (**B**) DNA sequencing identifies the c.298_304delACTCCTA *mafba* mutation lines. The 7-bp deletion is indicated with a black frame. (**C**) Relative expressions of *mafba* determined by qRT-PCR analysis in sibling, *mafba^+/−^*, and *mafba^−/−^* embryos at 4 dpf. The β-actin served as the endogenous control. Data are represented as mean ± SD; *, *p* < 0.05; **, *p* < 0.01. (**D**) Western blot analysis shows the similar protein expression of Mafba between siblings and *mafba^+/−^* embryos, but a significant decrease in *mafba^−/−^* mutants at 4 dpf. GAPDH was used as the internal control.

To elucidate the physiological role of Mafba in vivo, we generated *mafba* knockout zebrafish using CRISPR/Cas9 technology. The target sites were designed in the exon of *mafba-201* (NM_131015.3, NP_571090.2), in a sequence common to both transcripts. The detailed design of the *mafba* knockout is shown in Figure 1A. A 7-bp deletion mutation (c. 298_304delACTCCTA) would generate a premature termination codon and encode a truncated Mafba protein (p. Ser100Argfs*141). The del7 mutation may abolish the Mafba protein’s function, and so this zebrafish line was selected for our research (Figure 1B).

To further ensure the 7-bp deletion mutation, we verified the expression of *mafba* on the mRNA and protein levels. Firstly, semi-quantitative RT-PCR analysis showed that the expression levels of both transcripts (*mafba*-201, *mafba*-202) are significantly reduced in *mafba* mutant zebrafish (Figure S2). Secondly, qRT-PCR analysis showed that *mafba* mRNA expression decreased by 25% in *mafba^+/−^* embryos and decreased more significantly in *mafba^−/−^* mutant embryos at 4 days postfertilization (dpf) (Figure 1C). This suggests that the truncated *mafba* mRNAs are subject to nonsense-mediated decay. Thirdly, qRT-PCR analysis showed that the mRNA expressions of Maf homologues, such as large Maf (*mafba*, *mafbb*, *mafa*, *c-maf,* and *nrl*) and small Maf (*maff*, *mafk*, *mafga,* and *mafgb*), decrease significantly in the *mafba* mutants (Figure S3). We speculated that *mafba* mutants may have systemic non-specific effects that reduce the expressions of many genes, otherwise affecting the viability of some tissues. Additionally, the temporal and spatial expression of *mafba* was determined in sibling (Figure S4A–J) and *mafba^−/−^* mutants (Figure S4K–T) via whole-mount in situ hybridization (WISH). *Mafba* displayed a dynamic expression pattern throughout embryo development (Figure S4A–J). It emerged in bud-stage embryos (Figure S4C) and was then expressed throughout the embryos (Figure S4D–J). Noticeably, at 36 hpf, *mafba* was broadly expressed in the rhombomeres 5 and 6 [36], the inner ear, the eye, and the kidney (Figure S4F,G). Moreover, the expression pattern seen at 2 dpf was maintained until 5 dpf (Figure S4H–J). Semi-quantitative RT-PCR analysis showed that the expression of *mafba* was initiated at early developmental stages (6-somites stage) and then stabilized from 12 h postfertilization (hpf) to 6 dpf (Figure S4U). This is similar to the temporal expression pattern of siblings, shown in Figure S4A–J. WISH results showed that *mafba* can be detected in *mafba^−/−^* mutants, and that it displayed strong expression at the bud stage and 6-somites stage (Figure S4M,N). However, from 24 hpf onwards, *mafba* mRNA levels were dramatically decreased in *mafba^−/−^* embryos and were almost undetectable from 3 dpf (Figure S4O–T). Western blot analysis also showed that the Mafba protein’s expression was markedly decreased in the mutant line at 4 dpf, but did not completely disappear, likely due to the maternal deposit (Figure 1D). In addition, immunostaining showed that Mafba was localized in the rhombomere (r) and inner-ear region of the sibling at 2 dpf. However, almost no Mafba signals were detected in *mafba^−/−^* embryos (Figure S4V), suggesting its potential correlation with hindbrain and inner-ear development.

### 3.2. Depletion of Zebrafish mafba Results in Inner-Ear Defects and Hearing Loss

**Figure 2:**
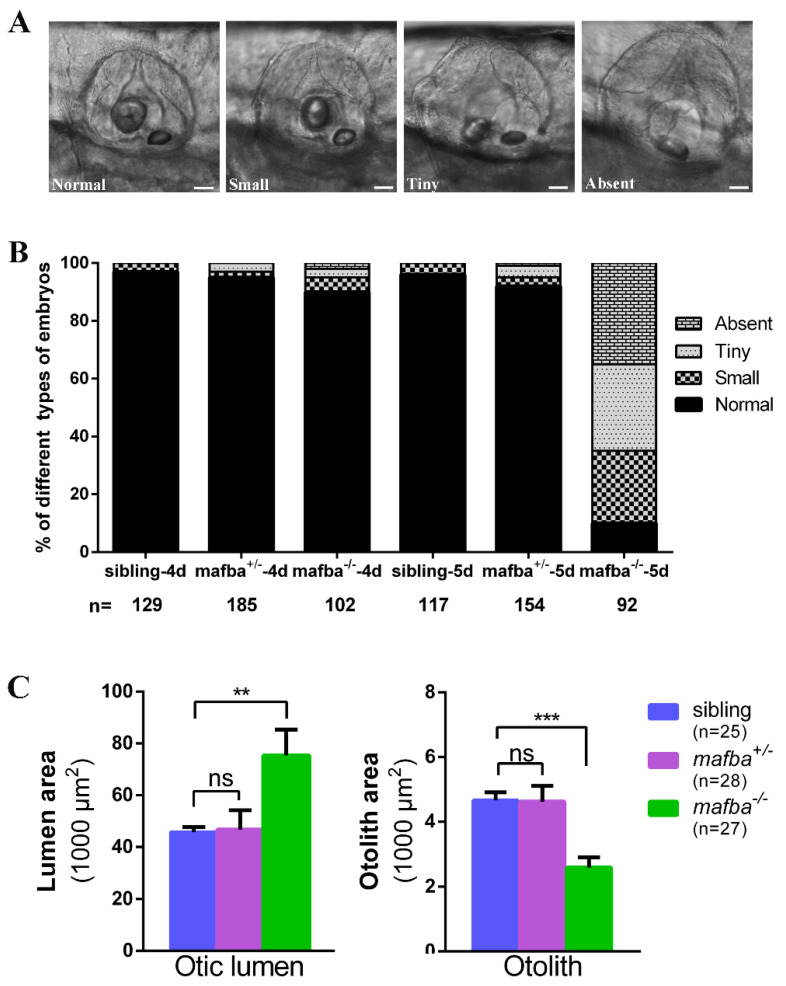
Deletion of *mafba* leads to inner-ear morphological defects. (**A**) The variable otolith sizes of *mafba^−/−^* mutant embryos at 5 dpf. According to the otolith sizes, mutant embryos were classified into four groups: normal, small, tiny, and absent. Scale bars: 40 µm. (**B**) Percentages of embryos in sibling and *mafba^−/−^* group at 4 dpf and 5 dpf; *n*, the number of observed embryos. (**C**) Statistical analysis of the otic lumen area and otolith area in different types of embryos at 5 dpf. Individual embryos were randomly picked from each type for statistical analysis. The otic lumen and otolith areas were measured with SPOT Advanced software (version 4.6) in the focal plane representing the maximal area. Data are represented as mean ± SD; ns, *p* > 0.05; **, *p* < 0.01; ***, *p* < 0.001.

To determine whether *mafba* is essential for inner-ear development in zebrafish, the phenotype of *mafba^−/−^* embryos was examined and compared with that of sibling embryos. The *mafba^−/−^* mutants were indistinguishable in gross morphology from the sibling and *mafba^+/−^* embryos in the inner ear at 4 dpf. Morphological defects of the inner ear abruptly occurred in the *mafba^−/−^* embryos at 5 dpf (Figure 2A,B). At 5 dpf, otoliths in the mutants showed varying degrees of size reduction. The otic defects of 5 dpf *mafba^−/−^* mutants included larger otocysts, thinner otic epithelia, and smaller or even eliminated otoliths (Figure 2A,C). According to the otolith size, we categorized the *mafba^−/−^* mutants into four groups: normal otoliths (accounting for 10.3%, *n* = 92), with otoliths comparable in size to the sibling embryos (on average 4662 ± 238 µm^2^); small otoliths (accounting for 24.4%), at 35–75% of the average otolith size of the siblings; tiny otoliths (30.1%), 10–34% of the average otolith size of the siblings; and absent otoliths (35.2%), at less than 10% of the otolith size of the siblings (Figure 2B). Less than 4% of the otic vesicles in sibling embryos (*n* = 117) could be categorized into the small group. Both the otic vesicle lumen and otolith sizes of zebrafish embryos were measured, and their areas were compared. The results further suggested the otic defect in mutants (Figure 2C). The inner-ear structures of *mafba^−/−^* mutants were almost destroyed. The mutation caused defects in inner-ears in a recessive mode.

**Figure 3:**
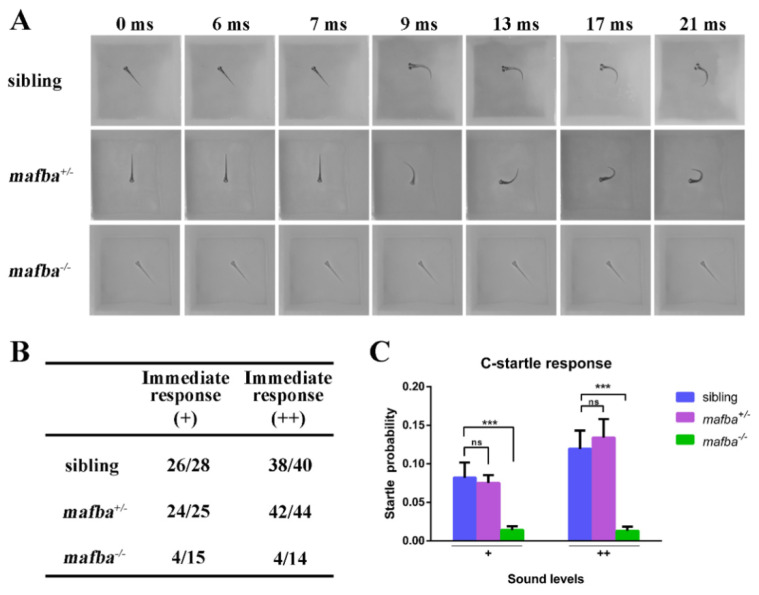
Hearing disability test for *mafba^−/−^* mutants at 5 dpf. (**A**) C-startle escape response of embryos of different genotypes in response to the sound stimuli at 500-Hz frequency and 10-ms duration. Typical C-startle escape response initiated within 9 ms after the sound stimulation. (**B**) Statistical data show the proportion of immediate responses in three genotypes at two different sound intensities. Data represent the number of immediate responses versus the number of total responses. ‘+’ and ‘++’ mean 20 dB and 40 dB, respectively. (**C**) The average C-startle response probability. For each group, 32 larvae were tested. Data are mean ± SD; ns, *p* > 0.05; ***, *p* < 0.001.

The inner ear plays crucial roles in zebrafish hearing and balance maintenance [53]. Due to the severe defects in the inner ears of *mafba^−/−^* mutants, we further investigated hearing abilities of the mutants. We assessed the fast escape reflex (named the C-shaped startle response) using near-field pure tone stimulation at two different levels of sound intensity [8]. The sibling and *mafba^+/−^* group larvae responded within 9 ms, but the *mafba^−/−^* larvae showed either a delayed response or no response at all at 5 dpf (Figure 3A).

For three genotypes, the numbers of immediate responses and delayed responses were recorded and are shown in Figure 3B. As described previously [54], we eliminated delayed responses and identified the percentage of successful C-shaped startle responses, and the result was that the probabilities of a C-startle response in sibling and *mafba^+/−^* embryos were indistinguishable and markedly higher than those of *mafba^−/−^* mutants (Figure 3C). These data support the idea that *mafba^−/−^* mutant zebrafish embryos display the hearing loss.

The survival rates of *mafba^−/−^* mutants are comparable with those of sibling and *mafba^+/−^* embryos up to 5 dpf. Most of the *mafba^−/−^* larvae showed inner-ear defects at 5 dpf. The survival rate of *mafba^−/−^* mutants decreased to 65% under normal feeding conditions, while the survival rate of *mafba^+/−^* embryos at 6 dpf was 92% (data not shown). This reduction may have been caused by the dietary deficiency resulting from the defective hearing and balance system [55].

### 3.3. Deficiency of mafba Does Not Affect Otic Patterning or Hair Cell Development

The early development of the inner ear appears be normal in *mafba^−/−^* mutants. The induction of the otic placode appeared as expected at 18 hpf. In order to comprehend the molecular function of *mafba* in ontogenesis, we assessed the expression levels of several marker genes involved in the inner-ear patterning structure. The expressions of patterning markers for medial otic vesicle (*pax2a*), dorsal otic vesicle (*dlx3b*), and ventral otic vesicle (*foxj1b*) showed no difference between the sibling and *mafba^−/−^* mutant embryos (Figure S6A–F). The expressions of the semicircular canal projections’ marker *udgh* (Figure S7G,H), the semicircular canal sensory cristae marker *bmp4* (Figure S7A,B), the utricular and saccular maculae marker *cahz* (Figure S7C,D), and the endolymphatic duct marker *foxi1* (Figure S7E,F) were also the same in the *mafba^−/−^* embryos and the sibling. In addition, live bright-field images of siblings (Figure S5A,C,E) and *mafba^−/−^* mutants (Figure S5B,D,F) showed that the inner-ear sizes were comparable at 54 hpf and the semicircular canal projections were forming normally. There was no obvious difference between the mutant and the sibling at 78 hpf in the otocysts, semicircular canal, utricle, and saccule. The organogenesis of the inner ear appeared to be normal until 102 hpf, suggesting the correct specification of early otic tissue.

The formation and mineralization of the otolith is a complex process that requires the orderly regulation and participation of many developmental processes, such as the secretion of matrix proteins [8], hair cell development, and endolymphatic fluid homeostasis [20], which are necessary for zebrafish otolith formation and growth. The WISH results showed that the expressions’ levels of the matrix protein genes *otomp* and *starmaker* (*stm*) at 2 dpf and 5 dpf in the sibling group were in accordance with those in *mafba^−/−^* embryos (Figure S8A–H). Then, we tested the otic hair cells, which play essential roles in the development of the inner ear. There was no significant difference between the *mafba^−/−^* and sibling embryos as stained by the hair cell marker *atp1b2b* at 24 hpf (Figure S9A). Immunostaining using the anti-Myo7a antibody indicated that the number of hair cells in *mafba^−/−^* and sibling embryos showed no difference in the utricular and saccular maculae at 36 and 48 hpf (Figure S9B,C). Phalloidin staining for stereociliary bundles and kinocilium growth labeled by Acetylated-α-Tubulin both suggested that the differentiation of the sensory cristae and maculae was normal in *mafba^−/−^* mutant embryos at 5 dpf (Figure S9D). In conclusion, these results implied that the patterning and structural specification of the otic vesicles, the differentiation and maturation of hair cells, and the growth of ciliary bundles were normal in the *mafba*-deficient embryos.

### 3.4. Knockout of mafba Suppresses the Proliferation of p63+ Epidermal Stem Cells and Reduces dlc+ Ionocyte Progenitor Cell Number

Mouse mafb plays an important role in regulating epidermal differentiation and homeostasis [56]. After confirming that matrix proteins and hair cell development were normal in the *mafba^−/−^* inner ear, the balance of ionocytes’ homeostasis was explored. Epidermal stem cells were marked with p63 and are known to produce both keratinocytes (dlc^−^p63^+^ cells) and ionocytes (dlc^+^p63^+^ cells). Whether *mafba* is expressed in the epidermis and affects the development of ionocytes in zebrafish embryos needs further investigation. The p63 expression was observed in the ventral ectoderm of 90% epiboly and bud-stage embryos [18], while the expression of Mafba occurred in the bud stage and 5-somites stage. The p63^+^Mafba^+^ colocalized cell number was high in the dorsal ectoderm region at the bud stage and 5-somites stage (Figure S10A). In addition, colocalization of Mafba and dlc was observed at the bud stage and 5-somites stages in the epidermal ionocyte region. Almost half of the dlc^+^ cells were positively stained with Mafba (Figure S10B). The colocalization of p63^+^Mafba^+^ and dlc^+^Mafba^+^ suggests that Mafba participates in the development of epidermal stem cells and ionocytes.

**Figure 4:**
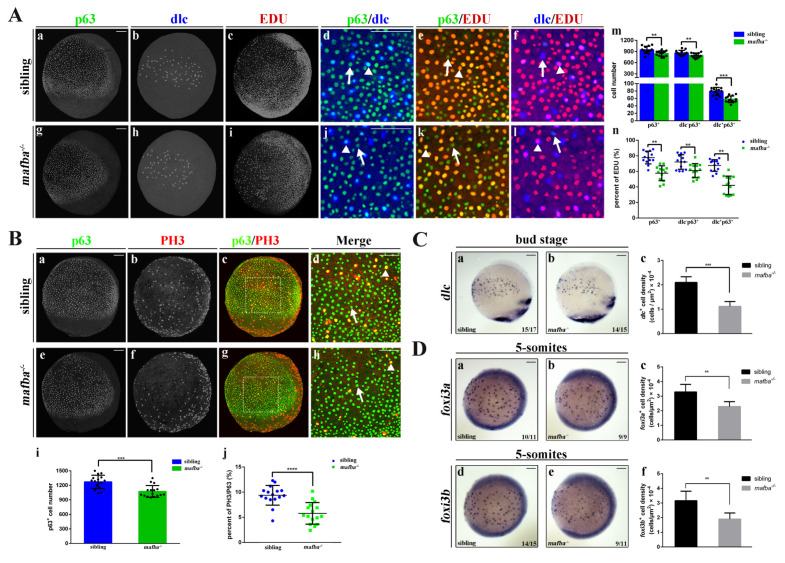
Knockout of *mafba* reduced the proliferation of epidermal stem cells and the *dlc^+^* ionocyte progenitor cell number. (**A**) Examples of p63 with dlc or p63 with EDU or dlc with EDU colocalized (arrowhead) or non-colocalized (arrow) cells between sibling and *mafba^−/−^* groups are shown in (**a**–**l**). The quantitative analyses of the p63^+^ (marker for epidermal stem cells), dlc^−^p63^+^ (marker for keratinocyte precursors), and dlc^+^p63^+^ (marker for ionocyte precursors) cells in each group at the bud stage are shown in (**m**). The quantitative analyses of p63^+^, dlc^−^p63^+,^ and dlc^+^p63^+^ cells colocalized with EDU (S-phase cells) in sibling and *mafba^−/−^* embryos at the bud stage are shown in (**n**). The *n* = 13 for each panel. Scale bars: 100 µm. (**B**) Double staining of p63 and pH3 (M-phase cells) in the siblings and *mafba^−/−^* group at the bud stage. Examples of p63 and pH3 colocalized (arrowhead) or non-colocalized (arrow) cells are shown. The quantitative analyses of p63^+^ cell and p63^+^ colocalized with pH3-positive cells of sibling and *mafba^−/−^* embryos at the bud stage are shown in (**i**) and (**j**), respectively. The *n* = 16 for each panel. Scale bars: 100 µm. (**C**) The *dlc^+^* ionocyte progenitors’ cell density was reduced in the *mafba^−/−^* group (**b**) as compared to the sibling group (**a**) at the bud stage. The quantitative analysis of *dlc^+^* ionocyte progenitors’ cell densities are shown in (**c**). (**D**) The cell density of *foxi3a^+^* and *foxi3b^+^* ionocyte progenitors are compared between the sibling (**a**,**d**) and *mafba^−/−^* group (**b**,**e**) at the 5-somites stage, respectively. Cell densities of *foxi3a^+^* and *foxi3b^+^* ionocyte progenitors are quantified in (**c**) and (**f**), respectively. Scale bars: 100 µm. Data are expressed as mean ± SD; **, *p* < 0.01; ***, *p* < 0.001; ****, *p* < 0.0001.

Mafb is widely known for regulating epidermal keratinocyte differentiation and epidermal homeostasis in mammals [27]. We speculated that zebrafish Mafba also modulates p63^+^ epidermal stem cell proliferation and dlc^+^ ionocyte progenitor differentiation. The p63^+^ epidermal stem cell number was reduced by 14.4% in *mafba^−/−^* mutants compared to the sibling group at the bud stage (Figure 4A(g,m)), and we also saw a 19.6% reduction in the proportion of p63^+^EDU^+^ epidermal stem cells in *mafba^−/−^* embryos. These results suggest that Mafba is essential to maintaining the proliferation rate of p63^+^ epidermal stem cells. The reduction in p63^+^ epidermal stem cell led to a decreased number of dlc^−^p63^+^ keratinocytes and dlc^+^p63^+^ ionocytes in *mafba^−/−^* mutant embryos at the bud stage (Figure 4A(g,h,j,m)). The percentage of EDU^+^ signals colocalized with dlc^−^p63^+^ and dlc^+^p63^+^ was significantly decreased in *mafba^−/−^* mutant embryos at the bud stage (Figure 4A(k,l,n)). A previous study suggested that *foxi3a* and *foxi3b* are the main regulators of epidermal ionocytes’ specification in zebrafish embryos [20]. At the bud stage, a prominent reduction in ionocyte progenitors was detected by dlc labeling (Figure 4C). A significant decrease in the densities of ionocyte progenitors expressing either *foxi3a* or *foxi3b* was observed in *mafba^−/−^* embryos at the 5-somites stage (Figure 4D). Mafba is, thus, essential to maintaining both the proliferation of p63^+^ epidermal stem cells and the cell numbers of dlc^−^p63^+^ keratinocytes and dlc^+^p63^+^ ionocytes.

**Figure 5:**
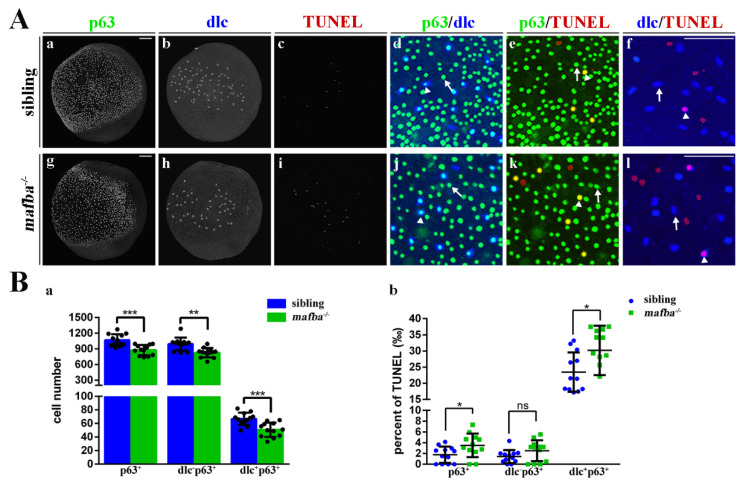
Increased apoptosis in the epidermal stem cells and *dlc^+^* ionocyte progenitor cells of *mafba^−/−^* zebrafish. (**A**) Examples of p63 with dlc, p63 with TUNEL, or dlc with TUNEL colocalized (arrowhead) or non-colocalized (arrow) cells are shown. (**B**) The quantitative analyses of p63^+^ cells, dlc^−^p63^+^ cells, and dlc^+^p63^+^ cells of sibling and *mafba^−/−^* embryos at the bud stage are shown in (**a**). The quantitative analysis of p63^+^, dlc^−^p63^+,^ and dlc^+^p63^+^ cell numbers colocalized with TUNEL in sibling and *mafba^−/−^* embryos at the bud stage are shown in (**b**). The *n* = 12 for each panel. Scale bars: 100 µm. Data are mean ± SD; ns, *p* > 0.05; *, *p* < 0.05; **, *p* < 0.01; ***, *p* < 0.001.

The significant reduction in p63^+^ epidermal stem cells in *mafba^−/−^* embryos suggests that these could be arrested in the cell cycle. To determine the cell cycle status of p63^+^ epidermal stem cells in *mafba^−/−^* embryos, EdU incorporation (labeling cells in S-phase) combined with the immunofluorescence of phospho-histone3 (labeling cells in M-phase) was performed at the bud stage. The p63^+^ epidermal stem cell number is also decreased in *mafba^−/−^* embryos (Figure 4B(e,i)) in accordance with the results shown in Figure 4A(m). Double immunostaining showed that the proportion of p63^+^pPH^+^ cells decreased significantly, by 3.6%, in *mafba^−/−^* mutant embryos (Figure 4B(g,h,j)). We thus speculated that the epidermal stem cells lacking Mafba may be arrested in G0/G1, and this may result in the increased apoptosis of these ionocyte progenitors. However, the TUNEL assays showed only a slight increase in apoptotic signal in the p63^+^ cells (by 1.73‰) and dlc^+^p63^+^ cells (by 6.71‰) in *mafba^−/−^* mutants compared to the sibling group, while no significant change was observed in dlc^−^p63^+^ cells (Figure 5). In summary, we confirmed a reduction in the proliferation of p63^+^ epidermal stem cells and the cell numbers of dlc^−^p63^+^ keratinocytes and dlc^+^p63^+^ ionocytes as well as the differentiation of the epidermal ionocytes’ progenitor in *mafba^−/−^* embryos.

### 3.5. Apoptosis of the Differentiated Epidermal Ionocytes Is Increased in mafba^−/−^ Embryos

**Figure 6:**
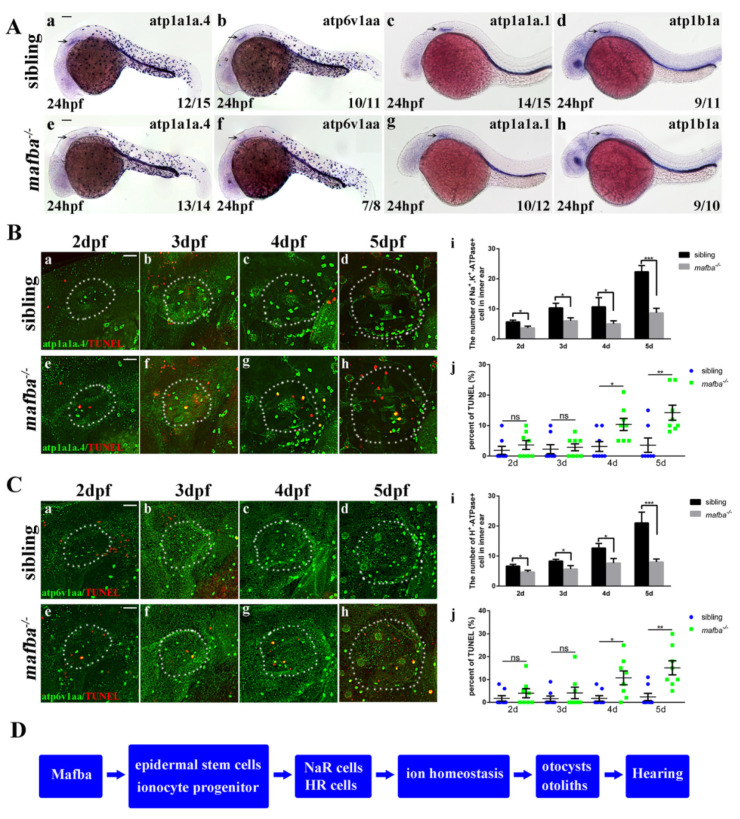
*Mafba* deprivation impaired ionocyte progenitor cell differentiation and triggered the apoptosis of NR and HR cells. (**A**) The in situ hybridization staining of markers for NR cells (*atp1a1a.4*, *atp1a1a.1*, *atp1b1a*) and for HR cells (*atp6v1aa*) at 24 hpf. The reductions in differentiated NR and HR cells in the inner-ear (arrow) region are shown. Scale bars: 100 µm. (**B**) Double immunostaining of atp1a1a.4 and TUNEL from 2 dpf to 5 dpf. The *n* = 8 for each panel. Scale bars: 20 µm (**a**–**h**). (**i**) The quantitative analysis of NR cells of the inner ear (the white, dotted, circled area) from 2 dpf to 5 dpf between sibling and *mafba^−/−^* mutants. (**j**) The percentage of the apoptosis in the NR cells from 2 dpf to 5 dpf indicated increased apoptosis at 4 dpf and 5 dpf in *mafba^−/−^* embryos’ inner-ear regions. (**C**) Double immunostaining of atp6v1aa and TUNEL from 2 dpf to 5 dpf. The *n* = 8 for each panel. Scale bars: 20 µm (**a**–**h**). (**i**) The quantitative analysis of HR cells of the inner-ear (the white, dotted, circled area) from 2 dpf to 5 dpf between sibling and *mafba^−/−^* mutants. (**j**) The quantification of the apoptosis percentage of NR cells from 2 dpf to 5 dpf indicates an increase at 4 dpf and 5 dpf in *mafba^−/−^* embryos’ inner-ear regions. Data are represented as mean ± SD; ns, *p* > 0.05; *, *p* < 0.05; **, *p* < 0.01; ***, *p* < 0.001. (**D**) A model of Mafba functions in zebrafish inner-ear development and hearing.

A previous study suggested that *foxi3a* and *foxi3b* control ionocyte progenitor specification into NR and HR cells through a positive feedback loop [24]. Ionocyte progenitors’ regulators, such as *dlc*, *foxi3a,* and *foxi3b*, were downregulated in *mafba^−/−^* mutant embryos. We, thus, wished to assess whether the formation of NR and HR cells is affected by deprivation of *mafba* expression. We performed WISH using relevant markers (*atp1a1a.1*, *atp1a1a.4,* and *atp1b1a* for NR cells, *atp6v1aa* for HR cells) to evaluate the differentiated epidermal ionocytes in the inner ear. At 24 hpf, the expressions of *atp1a1a.1*, *atp1a1a.4, atp1b1a,* and *atp6v1aa* in the *mafba^−/−^* embryos reduced markedly compared to those in the siblings (Figure 6A). Similarly, a significant reduction in atp1a1a.4 and atp6v1aa expression in the inner ear was observed in *mafba^−/−^* mutants from 2 dpf to 5 dpf (Figure 6B,C), respectively. Considering the dramatic decline in atp1a1a.4- and atp6v1aa-expressing cells in the inner ear at 5 dpf, we suspected that the apoptosis may also occur in *mafba* mutants. To validate this hypothesis, we measured the apoptosis of atp1a1a.4- and atp6v1aa-expression cells in *mafba^−/−^* embryos using TUNEL assays. There was a significant increase in apoptotic atp1a1a.4 and atp6v1aa cells’ signals in the inner-ear region in *mafba^−/−^* embryos compared with those in the siblings at 4 dpf and 5 dpf (Figure 6B(j),C(j)). The percentage of apoptotic cells with atp1a1a.4 and atp6v1aa at 5 dpf was notably higher than that at 4 dpf. The defective inner ear might result from the decreased ionocyte progenitors and increased apoptosis in NR and HR cells. In summary, Mafba is essential to the proliferation of the epidermal stem cells and ionocyte progenitors, which, in turn, determine the numbers of NR and HR cells and maintain ion homeostasis in zebrafish inner ear. The differentiated ionocyte progenitors provide a stable environment for the development of statoacoustic organs such as otoliths and otocysts, which are essential to the hearing and balance systems (Figure 6D). However, more research is needed to reveal the specific regulatory mechanism of inner-ear developmental defects.

### 3.6. Deprivation of mafba Activates p53 Apoptosis Pathway and Arrests the Cell Cycle in the G0/G1 Phase

**Figure 7:**
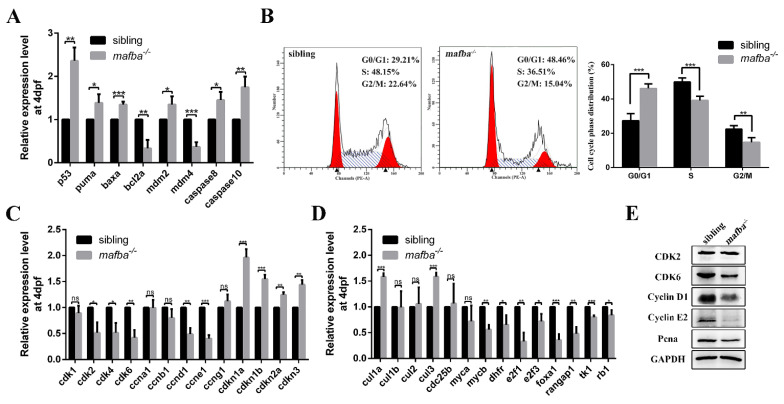
*Mafba* deficiency causes G0/G1 cell cycle arrest and activates the p53 pathway. (**A**) The upregulation of p53 pathway genes in *mafba* mutants at 4 dpf was detected by qRT-PCR. (**B**) The cell cycle phase distribution was performed by flow cytometry at 4 dpf. The proportion of sibling and *mafba^−/−^* embryos in the G0/G1, S, and G2/M phase cell numbers. The β-actin served as endogenous control. (**C**) Quantitative RT-PCR analysis of the primarily relevant cell cycle factors in sibling and *mafba^−/−^* mutant embryos at 4 dpf. (**D**) The key cell cycle factors controlling G1/S phase transition were analyzed via RT-qPCR at 4 dpf. (**E**) Western blot analysis of CDK2, CDK6, Cyclin D1, Cyclin E1, and Pcna at 4 dpf. GAPDH was used to normalize the protein. Data are mean ± SD; ns, *p* > 0.05; *, *p* < 0.05; **, *p* < 0.01; ***, *p* < 0.001 compared to the sibling control (by ANOVA followed by Dunnett’s multiple comparison).

In order to determine how Mafba deprivation triggers apoptosis, we quantified the expression levels of several apoptosis-related genes, including *caspase8*, *caspase10*, and *p53,* and its target genes in zebrafish embryos. The expressions of *p53* and its target genes *baxa*, *mdm2,* and *puma*, along with apoptosis-related genes *caspase10* and *caspase8*, showed significant increases in *mafba^−/−^* mutants at 4 dpf. In addition, the expressions of *mdm4* and *bcl2a*, typical inhibitors of *p53*, decreased significantly (Figure 7A). Based on the observation that the proliferation of epidermal stem cells was suppressed, the p53 pathway was activated, and the apoptosis of NR and HR cells was increased, we hypothesize that G0/G1 cell cycle arrest may also occur in *mafba* mutants. A previous study revealed that mouse Mafb promotes cell proliferation with detectable changes in cell cycle factors [57]. Cell cycle distribution was examined via flow cytometry. The proportions of G0/G1 phase cells increased significantly, while S and G2/M phase cells decreased in the *mafba^−/−^* mutant at 4 dpf (Figure 7B). The knockout of *mafba* led to G0/G1 cell cycle arrest, suggesting it affects cell cycle factors.

A number of cell cycle factors that control G1/S phase transition were analyzed via RT-qPCR. The expressions of *cdk2*, *cdk4*, *cdk6, cyclin d1* (*ccnd1*), and *cyclin e1* (*ccne1*) were downregulated. This agrees with the results that the protein levels of CDK6, Cyclin D1, and Cyclin E1 were lower in *mafba^−/−^* mutants than in sibling embryos at 4 dpf (Figure 7C,E). Meanwhile, the cycle regulators of the G1/S checkpoint, such as *cnkn1a* (*p21*), *cdkn1b* (*p27*), *cdkn3*, *cul1a,* and *cul3*, were upregulated, while *mycb*, *dhfr*, *e2f1*, *e2f3*, *foxa1*, *rangap1*, *tk1,* and *rb1* were downregulated in *mafba^−/−^* mutants at 4 dpf (Figure 7C,D). These results indicated that cell cycle arrest occurred between the G1 and S phases in the mutants. The reduced protein levels of Pcna also confirmed the arrest of G1/S phase transition. These results suggest that Mafba is essential for cell proliferation via the regulation of cell cycle factors levels.

### 3.7. Accumulation of DNA Damages in mafba^−/−^ Inner-Ear

**Figure 8:**
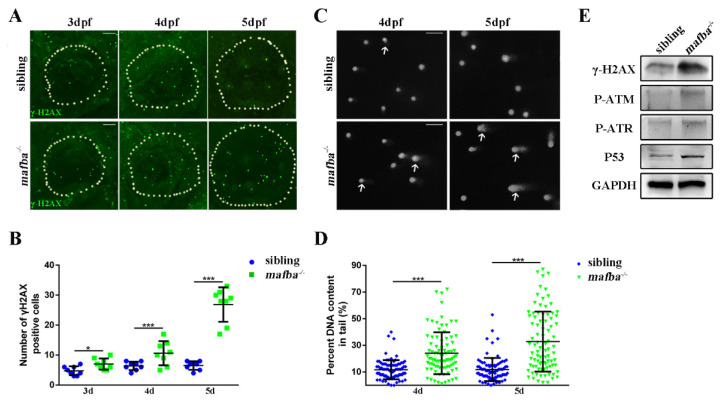
Analysis of the accumulation of DNA damage in *mafba^−/−^* mutants’ inner-ear. (**A**) Whole-mount immunofluorescence analysis using the anti-γH2AX antibody in siblings and *mafba^−/−^* inner-ear at 3, 4, and 5 dpf. The white, dotted circle represents the inner ear area. Scale bars: 20 µm. (**B**) Statistical analysis of the γH2AX-positive cells is shown in (**A**). (**C**) The alkaline comet assay showed increased DNA damage in *mafba^−/−^* embryos at 4 and 5 dpf. White arrows show DNA-damaged cells with single- or double-strand breaks. Scale bars: 10 µm. (**D**) Quantitative analysis of 88 cells from 10 embryos in siblings and *mafba^−/−^* group are shown. (**E**) Western blot analysis of γH2AX, P-ATM, P-ATR, and p53 in siblings and *mafba^−/−^* zebrafish at 4 dpf. GAPDH was used as the normalized protein control. Data are represented as mean ± SD; *, *p* < 0.05; ***, *p* < 0.001.

The apoptosis of the differentiated ionocytes may have resulted from the activation of the p53 pathway and/or cell cycle arrest at 4 dpf in *mafba^−/−^* inner ear. DNA damage may also occur in *mafba^−/−^* mutants, which would explain the apoptosis. The immunostaining of γH2AX indicated the DNA damage in sibling and *mafba^−/−^* inner ears at 3, 4, and 5 dpf (Figure 8A,B). The γH2AX-positive cells were observed in *mafba^−/−^* mutant inner ears at 3 dpf. The numbers of γH2AX-labeled cells in *mafba^−/−^* embryos inner ears increased dramatically at 4 and 5 dpf, compared to those in the sibling. DNA single/double-strand break levels were assessed directly via an alkaline comet assay [58]. We also detected more DNA breaking signals in *mafba^−/−^* mutants at 4 and 5 dpf (Figure 8C,D). Meanwhile, Western blot analysis also showed that the expression levels of γH2AX, P-ATM, P-ATR, and p53 accumulated gradually in *mafba^−/−^* embryos (Figure 8E). These results suggest that Mafba is essential for preventing DNA damage and maintaining genomic stability in differentiated ionocytes.

## 4. Discussion

In the present study, we characterized a zebrafish *mafba* knockout line generated using CRISPR/Cas9 technology. The inner ears of mutants showed specific defects, including enlarged otocysts, small or absent otoliths, and insensitivity to sound stimulation, whereas matrix protein expression and hair cells’ development appeared to be normal. Genetically, *mafba* plays a positive role in epidermal stem cells’ proliferation and can also promote the differentiation of the ionocyte progenitor in zebrafish inner ears. It also takes part in the differentiation of NR and HR cells, which are necessary for the ion homeostasis and epidermis differentiation.

The mouse *kreisler*/*mafb* is a member of the Maf transcription factor family. When mutated, it is identified as the causative gene of physiological defects resulting in segmentation abnormalities in the caudal hindbrain and defective inner-ear development [59]. In various vertebrates (including zebrafish), there are two paralogous othologues of mammalian *mafb*, i.e., *mafba* and *mafbb* [60,61]. Progressive syndromic deafness caused by heterozygous loss-of-function *Mafb* mutations was identified in a large family, though severity and age of onset differed among individuals. The mutation was located in the DNA-binding domain [33,34]. Researchers have been unable to characterize this inner-ear defect in mice because mutations in the mafb locus are lethal in the early postnatal period [62]. The locus of the mutation in our *mafba* zebrafish line is similar to that of the 0819 mutation in human MAFB [34], which explains why *mafba^+/−^* mutants have no effect on inner-ear defects. A mouse model with conditional knockout *Mafb* in the otic epithelial cells may help to illustrate the function of *mafba* in mammalian inner-ear development. Previous studies have shown that Mafb not only promoted the differentiation of otic epithelial cells, but also acted in the gene expression program that positively regulates epidermal keratinocyte differentiation [27]. Our research revealed that zebrafish *mafba^−/−^* mutant embryos exhibit inner-ear defects, with variable expressivity between individuals. The mutants with the more severe phenotypes possess bigger otocysts and smaller otoliths. Therefore, the mutant zebrafish *mafba^−/−^* line is the optimum animal model for progressive hearing loss in humans caused by *mafb* mutation, and can be applied to further study the mechanisms underlying *mafb*-associated inner-ear developmental defects.

Mafba plays crucial roles in various tissues and developmental stages. Based on a previous study and our research, zebrafish *mafba* is expressed in the otic vesicles, hindbrain, renal system, visual system, and other tissues during embryogenesis [36,63]. Developmental abnormalities in the inner ear were first detected in *mafba^−/−^* mutants at 5 dpf. Human progressive hearing loss caused by Mafb mutation is syndromic with other detectable defects, such as atopic dermatitis (AD), psoriasis vulgaris [27], aberrant extraocular muscle innervation, MCTO, and focal segmental glomerulosclerosis (FSGS) with DRS [33,34]. Zebrafish Mafba and human Mafb share a highly conserved amino acid sequence. The similarity in the inner-ear phenotype between zebrafish and human *mafb*-deficient individuals implies that *mafba* may be essential to inner-ear development and the maintenance of hearing capacity. In addition, *mafba^−/−^* mutants need to be examined at later stages of development to determine whether other tissues/organs are affected.

It is widely known that apical junctional complex proteins (AJC), extracellular matrix (ECM), and ion channels in the otic epithelial/epiderma cells act as barriers and are important to homeostasis maintenance, which is essential to normal primary inner-ear formation and subsequent development. The adhesion class G protein-coupled receptor (Gpr126) acts through a cAMP-mediated pathway to control the outgrowth and adhesion of canal projections in the zebrafish inner ear via the regulation of ECM gene expression [64]. The secretion of ECM molecules drives the growth of the epithelial projections and regulates the morphogenesis of the semicircular canals [65]. Regulated fluid secretion is crucial for many organs. The loss of chloride channel cystic fibrosis transmembrane conductance regulator (CFTR)-mediated fluid secretion impairs Kupffer’s vesicle (KV) lumen expansion, leading to defects in organ laterality [66]. Interestingly, a previous study showed that fluid accumulation increases hydrostatic pressure in the lumen. Consequently, the stress passes into the epithelium and expands the otic vesicle. In turn, this pressure inhibits fluid transport into the lumen. This negative feedback between pressure and transport allows the otic vesicle to adjust its growth rate, resulting in otic vesicle size variations [67]. A previous study showed that mouse *Kreisler*/*Mafb* and zebrafish *valentino*/*mafba* mutants display early inner-ear defects that are related to abnormalities in the hindbrain development [36,68]. Facial motor neurons will fail to migrate through r5/r6 and complete caudal migration in zebrafish embryos with *mafba* deficiencies. [69]. The expression of genes in r5/r6 is regulated by RA, FGF, *hnf1ba,* and *valentino* (*mafba*); any losses of these factors will abolish r5/r6 gene expression [70]. Mafba is also essential to abducens’ motor neuron development in zebrafish [71]. It has also been shown that the expression of *fgf3* in the hindbrain rhombomere is essential to the dorsal otic vesicle pattern in *valentino mutant zebrafish*, and its primary role is to maintain and focus the expression of the dorsal inner-ear gene that is induced by WNT signals [72]. Both of these reports imply that the otic vesicle pattern is regulated or influenced by hindbrain development. Interestingly, other researchers have provided evidence that *fgf3* is not sufficient to guide otic regionalization [35]. However, both of these studies focused on early inner-ear defects caused by cascading effects of the external environment, such as abnormal hindbrain development. Whether there is any direct internal mechanism responsible for the abnormal inner-ear structure is yet to be determined.

The transcription factor *Mafb* plays a crucial role in epidermal keratinocyte differentiation in the mammalian epidermis by regulating downstream key transcription factors, such as *grhl3*, *znf750*, *klf4*, and *prdm1* [73,74] The ectopic expression of Mafb in basal keratinocytes results in excessive proliferation and disturbs epidermal homeostasis [56]. The terrestrial vertebrate epidermis is a stratified epithelium consisting of distinct layers of keratinocytes, which build a functional osmotic barrier that prevents dehydration. In contrast, the fish epidermis is constituted of keratinocytes and ionocytes, which transport ions and acid-base substances to maintain the homeostasis of bodily fluids [75]. Most, if not all, studies of Mafb have focused on the homeostasis of epidermal keratinocytes and pay no attention to the ionocytes. In zebrafish embryos, epidermal stem cells specify and differentiate into ionocytes and keratinocytes during the bud stage, which is controlled by the Dlc-Notch-mediated lateral inhibition [18,75]. Our data revealed that Mafba helps to maintain the ionocyte progenitor population by regulating epidermal stem cell proliferation, resulting in fewer stem cells and a decreasing number of differentiated ionocyte progenitors, and then decreases NR and HR cells in the inner-ear region. Finally, the loss of homeostasis could impede the volume control of the otocysts and the growth of otoliths. Collectively, these defects lead to hearing and balance problems (Figure 6D). Previous studies have shown that *klf4*, which serves as a downstream regulator of *mafba*, maintains the ionocyte progenitor population via *dlc*-mediated lateral inhibition [18]. Taken together, our results clarify the novel and key role of *mafba* in the maintenance of ionocytes and ion homeostasis during zebrafish embryogenesis.

Interestingly, unlike in mice [37], the apoptosis of NR and HR cells increases markedly in *mafba^−/−^* mutant inner ears compared with those of sibling embryos from 4 dpf. The discrepancy between mice and zebrafish phenotypes may stem from differences between species or it might also be attributed to diversities between ionocytes and other cells. The reduction in NR and HR cells was due to cell apoptosis, and this led to inner-ear ion homeostasis imbalances, which eventually resulted in the *mafba^−/−^* mutant inner-ear defects at 5 dpf. Follow-up studies identified that the activation of p53-mediated apoptosis and cell cycle arrest at the G0/G1 phase in *mafba^−/−^* embryos at 4 dpf and DNA damage appeared in the inner-ear area from 3 dpf to 5 dpf. DNA damage is a relatively common event that leads to the activation of DNA damage checkpoints, such as ATM and ATR. These signal transducers activate p53 and deactivate cyclin-dependent kinases to inhibit cell cycle progression [76]. These results imply that DNA damage is the major factor causing p53 activation in response to zebrafish *mafba* deletion. Our results show that *mafba* depletion leads to a reduction in CDK6 protein level and transcriptional activity, accompanied with an accumulation of *p53* mRNA and protein expression, which may be the mechanism of cell cycle arrest. Previous research has also shown that *mafb* deprivation destroys direct downstream regulator cyclin-dependent kinase 6 (CDK6) transcription and impedes clinical colorectal cancer (CRC) cell proliferation, as a result of the cell cycle arrest at the G0/G1 phase [37]. The specific deletion of *mafb* suppresses nasopharyngeal carcinoma cell (NCC) proliferation [77]. Overexpression of *mafb* enhances cell foci formation and increases cyclin B1 and D2 expression [40]. These studies suggest that DNA damage, p53 pathway activation, and the impairment of cell proliferation are responsible for the ion channel homeostasis imbalances in *mafba^−/−^* mutant inner ears. Aside from the ion channels, junction proteins are important for the homeostasis maintenance and inner-ear development [8,9]. Therefore, it is expected that *mafba* will exert its function on inner-ear development through the regulation of the junction proteins’ factor. Further studies may focus on identifying the downstream targets among these candidates in order to clarify the specific mechanism of zebrafish *mafba^−/−^* mutant inner-ear defects.

In summary, we established a *mafba* knockout zebrafish line displaying auditory impairment for the first time. We elucidated the roles of Mafba in maintaining ion channel homeostasis in the zebrafish inner ear via the control of the proliferation of the ionocyte progenitor and the populations of differentiated NR and HR cells. This study provides novel insights into the inner-ear pathogenic mechanisms of Mafba and offers an ideal model for identifying new therapeutic interventions for inner-ear defects.

## Data Availability

The data presented in this study are available on request from the corresponding author.

## Supplementary Materials

The following are available online at https://www.mdpi.com/article/10.3390/biomedicines9111699/s1. Figure S1: The domain features and homologous comparison of the transcription factor Mafb among mammals and zebrafish. Figure S2: The *mafba* mRNA levels were detected in 4-dpf sibling and *mafba* mutant zebrafish by qRT-PCR. Figure S3. Relative expressions of large maf (*mafba*, *mafbb*, *mafa*, *c-maf,* and *nrl*) and small maf (*maff*, *mafk*, *mafga,* and *mafgb*) were determined by qRT-PCR analysis in sibling, *mafba^+/−,^* and *mafba^−/−^* embryos at 4 dpf. Figure S4: Expression of the zebrafish *mafba* gene during embryonic development in siblings and its corresponding homozygous mutants. Figure S5: Early patterning of the inner-ear appears normal in *mafba^−/−^* mutants. Figure S6: Early patterning of the otic vesicle appears normal in *mafba^−/−^* mutants. Figure S7: The expression of otic specification-related markers was normal in *mafba^−/−^* mutant embryos. Figure S8: The expression of two otic matrix protein markers was normal in *mafba^−/−^* mutant embryos at 2 and 5 dpf. Figure S9: Deficiency of *mafba* does not affect hair cell development. Figure S10: Mafba protein expression pattern and colocalization of Mafba/p63 and Mafba/dlc during late-gastrulation and early-somite stages. Table S1: Primers used for Cas9 target design, WISH probes construction, genotype identification, and qRT-PCR.
