# Supplementary material for: Knockout of mafba Causes Inner-Ear Developmental Defects in Zebrafish via the Impairment of Proliferation and Differentiation of Ionocyte Progenitor Cells
Source: Biomedicines. 2021 Nov 16;9(11):1699. doi: 10.3390/biomedicines9111699 (PMC8616026; doi:10.3390/biomedicines9111699)
Supplement: Supplementary file 1 [file biomedicines-09-01699-s001.zip › biomedicines-1357249-supplementary.pdf]

Supplementary Files

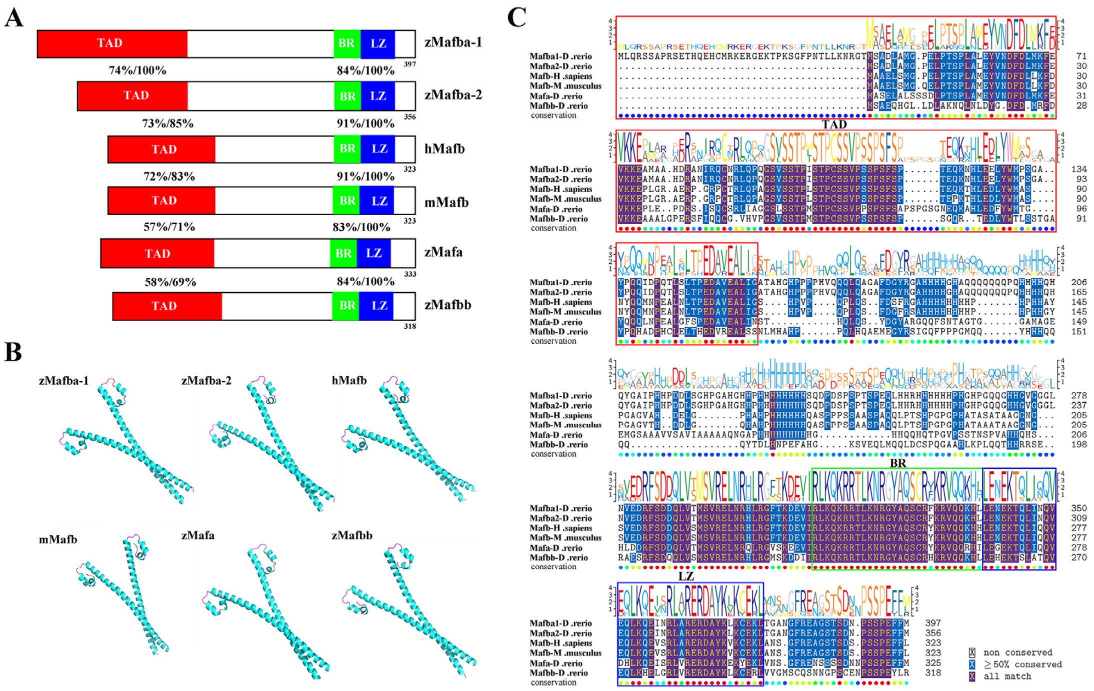

Figure S1. The domain features and homologous comparison of the transcription factor Mafb among mammals and zebrafish. (**A**) Schematic representation of the three functional domains of zebrafish, mouse and human Mafb proteins, and the Mafa of zebrafish, respectively. The percentage of identity/similarity was indicated for the transactivation domain (TAD), the basic region (BR) and the leucine zipper domain (LZ). (**B**) 3D protein model of the (**A**) were shown in (**B**). (**C**) The amino acid sequence alignment of Mafb and Mafa domains in various species. The red box represented the transactivation domain (TAD), green box and blue box represented he basic region (BR) and the leucine zipper domain (LZ), respectively.

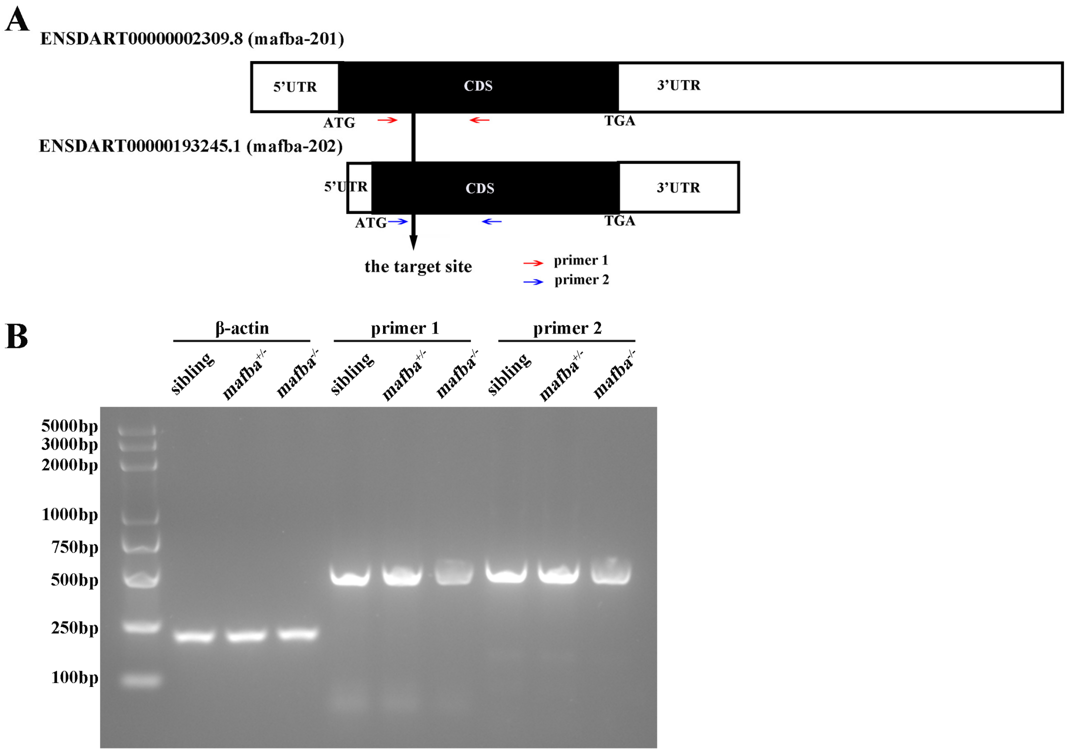

Figure S2. The *mafba* mRNA levels were detected in 4 dpf sibling and *mafba* mutant zebrafish by RT-PCR. (**A**) Location of the two primer pairs used for RT-PCR experiments. (**B**) Semi-RT-PCR analysis shows the relative expression of *mafba* in sibling, *mafba*$^{+/-}$ and *mafba*$^{-/-}$ embryos at 4 dpf. β-actin was served as endogenous control.

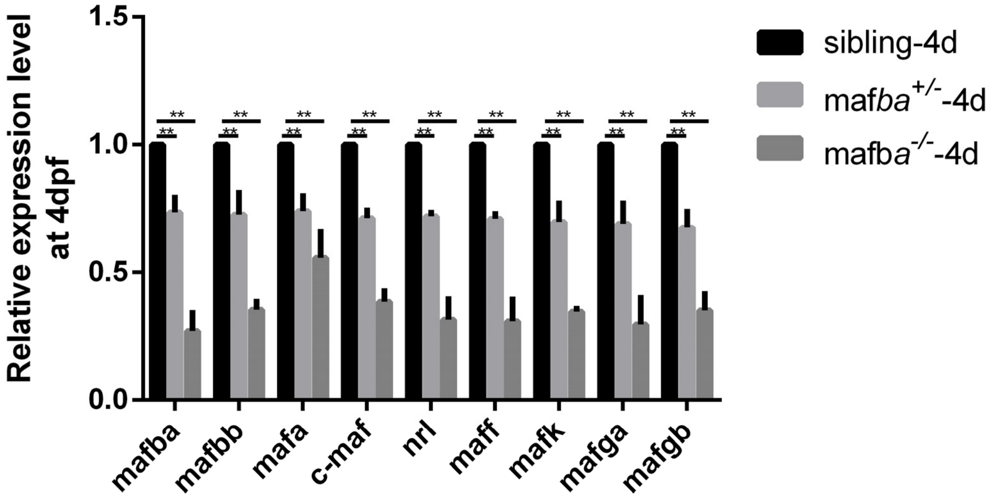

Figure S3. Relative expressions of large maf (*mafba*, *mafbb*, *mafa*, *c-maf* and *nrl*) and small maf (*maff*, *mafk*, *mafga* and *mafgb*) were determined by qRT-PCR analysis in sibling, *mafba*$^{+/-}$ and *mafba*$^{-/-}$ embryos at 4 dpf. β-actin was served as endogenous control. Data are represented as mean ± SD in each bar. *, $p < 0.05$; **, $p < 0.01$.

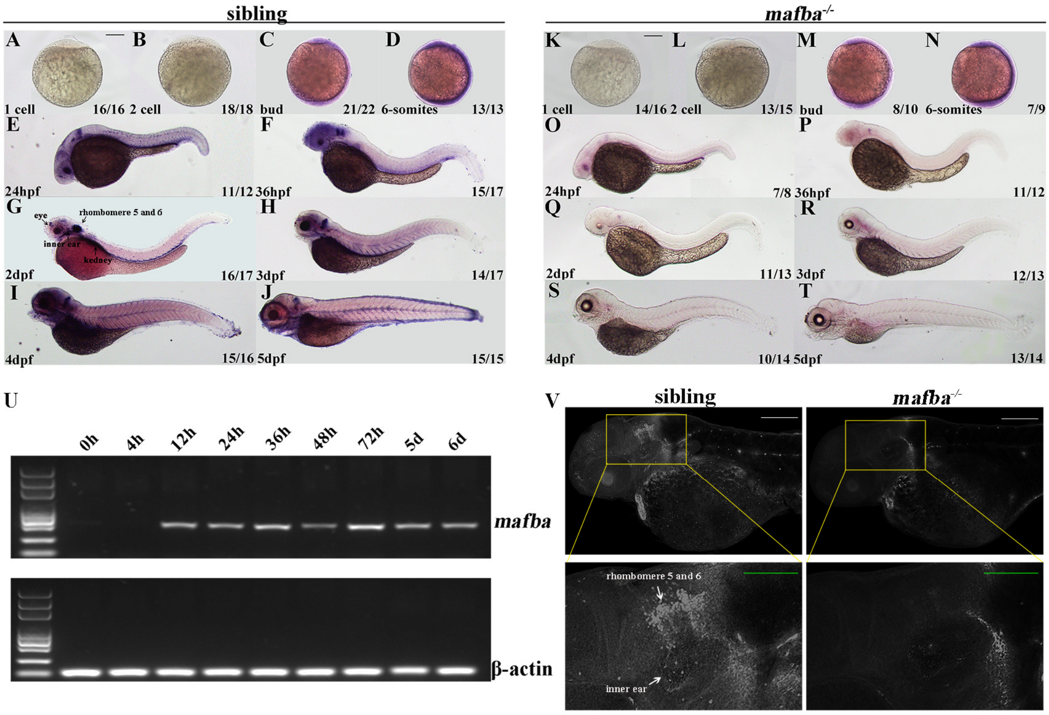

Figure S4. Expression of the zebrafish *mafba* gene during embryonic development in siblings and its corresponding homozygous mutants. Whole-mount *in situ* hybridization analysis of *mafba* gene expression in siblings (**A**-**J**) or *mafba*$^{-/-}$ mutants (**K**-**T**) at the developmental time-points indicated. Numbers at the bottom right indicate the number of embryos with similar staining pattern among all embryos examined. Scale bar: 200 μm. (**U**) RT-PCR amplification from different embryonic developmental stages. β-actin was used as an internal control. (**V**) Immunostaining of the siblings and *mafba*$^{-/-}$ mutants zebrafish embryos using the antibody against zebrafish Mafba at 2 dpf. Dots indicate Mafba expressed cells. $n = 10$ for each panel. White scale bar: 200 μm, green scale bar: 100 μm.

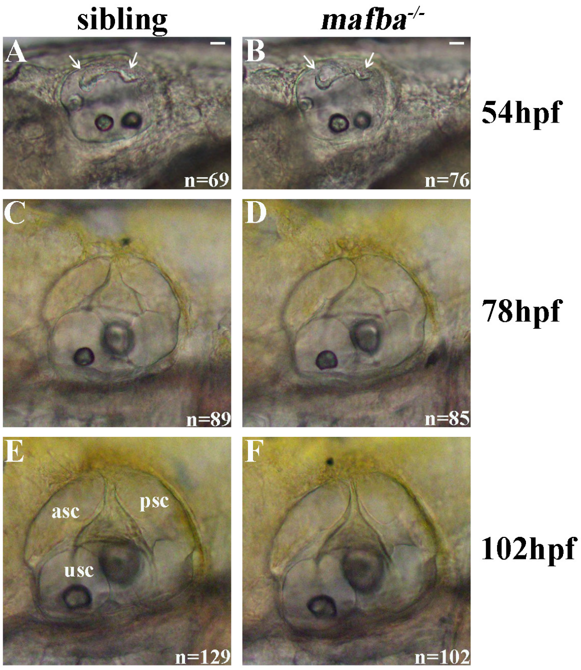

Figure S5. Early patterning of the inner ear appears normal in *mafba*$^{-/-}$ mutants. Live embryo pictures of siblings (**A**, **C**, **E**) and *mafba*$^{-/-}$ mutants (**B**, **D**, **F**) show that the inner ear sizes are comparable at 54 hpf and that the semicircular canal projections are forming normally (white arrow). And, no difference is observed between sibling and *mafba*$^{-/-}$ mutants at 78 hpf and 102hpf. asc, anterior semicircular canal; psc, posterior semicircular canal; usc, utriculosaccular chamber. Scale bar: 20 μm.

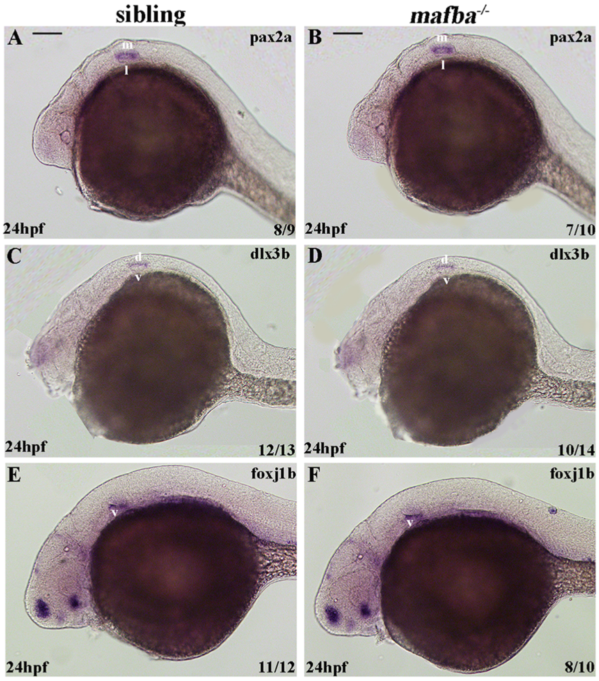

Figure S6. Early patterning of the otic vesicle appears normal in *mafba*$^{-/-}$ mutants. Whole-mount in situ hybridization shows that the expression of early patterning markers in the otic vesicle is indistinguishable between siblings and *mafba*$^{-/-}$ mutants. Normal expression is seen for *dlx3b* dorsally (**A**, **B**), *pax2a* medially (**C**, **D**) and *foxj1b* ventrally (**E**, **F**) at 24 hpf. m, medial; l, lateral; d, dorsal; v, ventral; Scale bar: 100 μm.

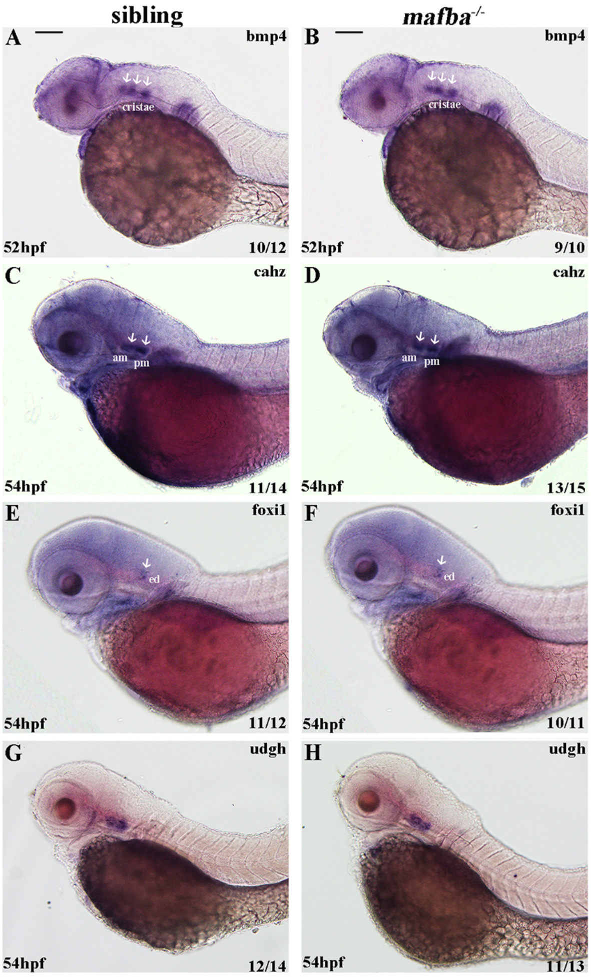

Figure S7. The expression of otic specification related markers was normal in *mafba*$^{-/-}$ mutant embryos. *In situ* hybridization was performed using *bmp4*, *cahz*, *foxi1* and *udgh* probes. *bmp4* and *cahz* mark the cristae (**A**, **B**, white arrow) and maculae (**C**, **D**, white arrow), respectively. The endolymphatic duct (ed), marked by expression of *foxi1* (E, F, white arrow), appears to evaginate normally. The semicircular canal projections marked by *udgh* also form normally (**G**, **H**). AM, anterior macula; PM, posterior macula; Scale bar: 100 μm.

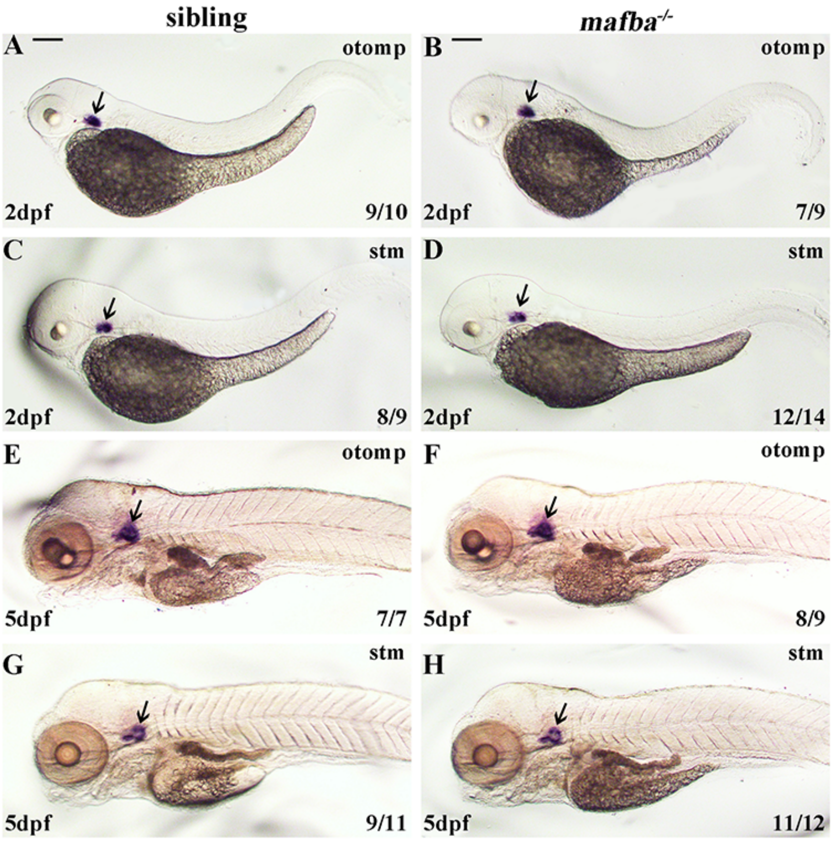

Figure S8. The expression of two otic matrix protein markers was normal in *mafba*$^{-/-}$ mutant embryos at 2 and 5 dpf. The signal points were pointed by black arrows. *In situ* hybridization was performed using *otomp* (**A**, **B**, **E**, **F**) and *stm* (**C**, **D**, **G**, **H**) probe, respectively. Scale bar: 200 μm.

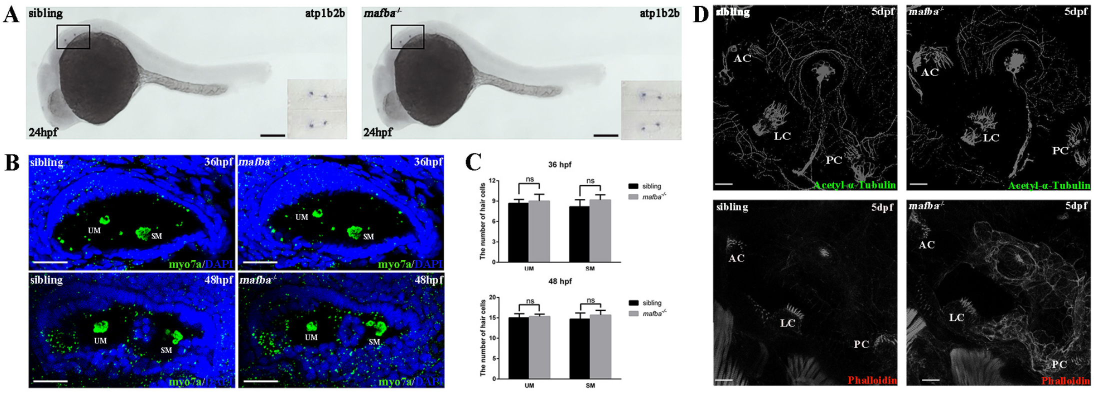

Figure S9. Deficiency of *mafba* does not affect hair cell development. (**A**) The expression of *atp1b2b* in hair cells at 24 hpf is normal in *mafba*$^{-/-}$ mutants, which is detected by in situ hybridization. $n = 12$ for sibling and $n = 11$ for *mafba*$^{-/-}$ mutants group. Scale bar: 200 μm. (**B**) Immunostaining of hair cells by anti-Myo7a antibody at 36 and 48 hpf. Red color indicates myo7a expressed cells. $n = 10$ for each panel. UM, utricular maculae; SM, saccular maculae; Scale bar: 20 μm. (**C**) The quantitative analysis of hair cell numbers in the utricular and saccular maculae at 36 hpf and 48 hpf shown in (**B**). Data are represented as mean ± SD. ns, $p > 0.05$ by one-way ANOVA. (**D**) Immunofluorescence using anti-acetylated tubulin antibody shows normal kinocilia in mutants at 5 dpf. Phalloidin staining showed normal stereociliary bundles in mutants at 5 dpf. $n = 12$ for each panel. AC, anterior crista; LC, lateral crista; PC, posterior crista; Scale bars: 20 μm.

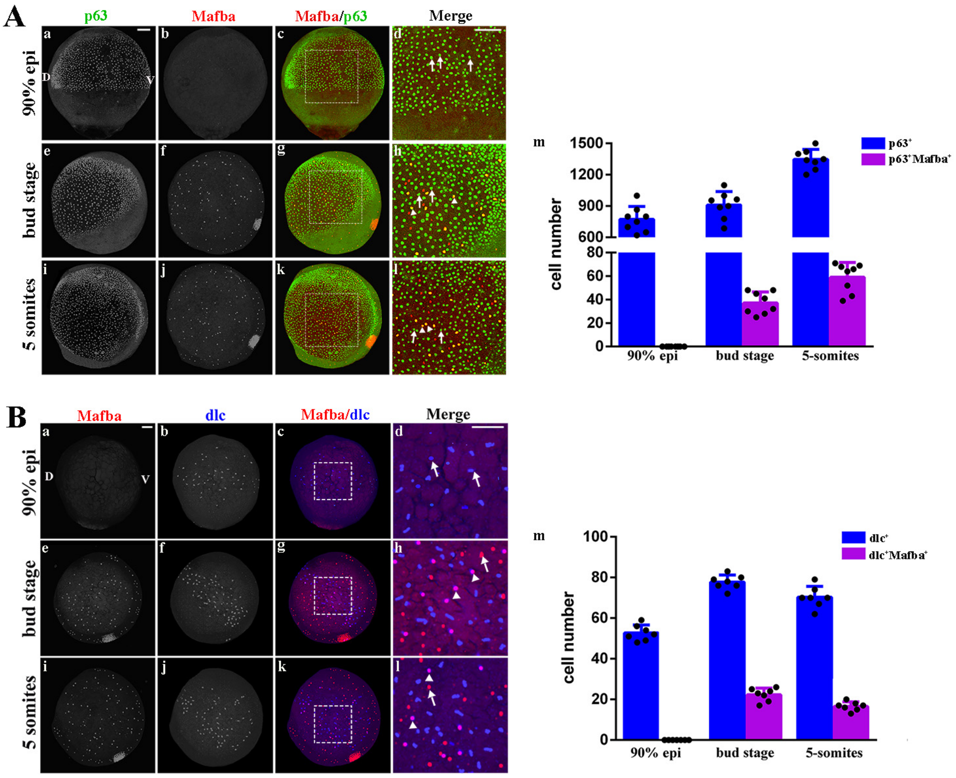

Figure S10. Mafba protein expression pattern and colocalization of Mafba/p63 and Mafba/dlc during late gastrulation and early somite stages. (**A**) Images of Mafba protein expression during different embryonic developmental stages are shown (b, f, j), and the p63 expression during different embryonic developmental stages are shown (a, e, i). Colocalization of Mafba and p63 is detected in ventral ectoderm of embryos at various stages, including 90% epiboly, bud and 5s (c, g, k). Enlargements of Mafba and p63 merged images (d, h, l) are shown from the areas (c, g, k). *p63*$^+$ epidermal stem cell number and p63$^+$Mafba$^+$ cell number at indicated embryonic stages are shown in (m). n = 8 for each panel. Scale bars: 100 µm. (**B**) Images of Mafba protein expression during different embryonic developmental stages are shown (a, e, i), and the dlc protein expression during different embryonic developmental stages are shown (b, f, j). Colocalization of Mafba and dlc was detected in ventral ectoderm of embryos at various stages, including 90% epiboly, bud and 5s (c, g, k). Enlargements of Mafba and dlc merged images (d, h, l) are shown from the (c, g, k). *dlc*$^+$ epidermal stem cell number and dlc$^+$Mafba$^+$ cell number at indicated embryonic stages is shown in (m). n = 7 for each panel. Scale bars: 100 µm.

Table S1. Primers used for Cas9 target design, WISH probes construction, genotype identification, and qRT-PCR.

Terms	Primer names	Forward	Reverse
**Cas9 target**	*mafba*	TGCTCGGGCGAGGTAGGAGAAGG	
**WISH Probes**	*mafba*	TGGAGGAGCTGTACTGGATGC	CGAGGTAGGAGAAGGGCTGTC
	pax2a	CTCCATATCGTACCCGTCTGAA	CGAACACAAACCATCAGCGAAA
	dlx3b	TCTCCCAGAGTCATCGGCTA	CGTTTACCATCCGCACTTC
	foxj1b	GAATGCCTGCCACAAACTT	TGAGAACATCCCCTCGTAA
	bmp4	CCTTGCTGTGGAGGTTGTA	CGTGATTGGTGGAGTTGAG
	cahz	GAAGTATGACCCAGCCACC	GTCTGTCTGCCCTTTGATT
	foxi1	CCAAACTGCGAGAAGATGT	ACTGTTGTTGTGCGATGCT
	udgh	ACACCTGGTCCTCTGAACT	CGTCACCTTCTTACCGATG
	atp1b2b	TTTCATCCAGGAGGACAGCG	CCAACTTTGTATTTCTTCGCACC
	otomp	CACCCAGGGATGAGGACTA	GCACATCAGGCTGCCCATA
	stm	CTGACAGTGCATCGGTAGAGG	GCATAGAAGTTTTCCTGCCATC
	dlc	GGCAGTTGCAATGACCAGGA	ACCTTAAATGGGCCACCAGG
	foxi3a	TGACAGCGATCCAGGAAAAGG	TACTCAGGCACCGCAATGAG

	foxi3b	CTGGAGGGGAATTGAGCTGG	TGCCCACAGAACTCAAAGGAG
	atp1a1a.4	TCCTCCCCTCCGATTTAGT	CACGGTTTGAGCGGATACA
	atp6v1aa	ACGCAGAGCATCTACATCCC	ATGGCAGTGGTTCCTCCTTG
	atp1a1a.1	TACCTTTGGGCACCGTCA	TCTATCTTCCCAGCCAACG
	atp1b1a	AGCCCACATACCAGGACAGG	ACTCGCCCAGCCATTCTC
**Genotype Identification**	*mafba(wt)*	GTCCTCCACTCCTATCAGCA	CGCCGTACTGGTGTTGATGGTGAT
	mafba(mt)	GGCTCCGTGTCCTCCTCAGC	CGCCGTACTGGTGTTGATGGTGAT
**RT-PCR**	*mafba-201*	CCCGCTGGCGTTGGAATATGTC	CGCCGTACTGGTGTTGATGGTGAT
	mafba-202	CGAGGTAAAGAAAGAGGCCATGG	GTGGTGGTGGTGATGGTGGGGGTG
	cdk1	GGCAGGAATAAAACCACAGGAC	TCAGCACATCTAGCAGGCGTA
	cdk2	AAGAGTTTCAGTCGCCCGTT	GGAACACCTTCAGTTTCCGTAT
	cdk4	ACGGTTACGGAGGATCAGCA	CACGTCCATCAGCCTGACGAT
	cdk6	CGGCAAATCACTCCTACTTCGTTTC	CGAAAGGAAAACCTGACGGAC
	ccna1	GGCCAAAGAATTGTGCTCGG	ATCCATAAATGACCCTGAGCC
	ccnb1	TGTGGTGACTGGACCCCTAC	GCAGCACACAGTCGTTAAGT
	ccnc1	TCCTCCTAACAGCGAGACTG	GAGGTGGAGTCTGCCTTGATT
	ccnd1	AAACACGCCCAGACCTTTGT	ATCGCAGACAGTCAGGGTC
	ccne1	AGCAGACGTGGCTATTCATT	GAAGAGGGGTAGAGCGTGTG
	ccng1	CATCTCTAAAAGAGGCTCTAGATGG	CACACAAACCAGGTCTCCAG
	cdkn1a	TCCAGCTTCAGGTGTTCCTCA	TGAACGTAGGATCCGCTTGT
	cdkn1b	AAGCTCCTGTCTCGACTCATC	TTATTCCCCCTCCTCGCTCC
	cdkn2a	GCGTTGAACTGATTGTTTTCG	GTTACCCATCATCATCACCTGTAT
	cdkn3	ATGTCCGCGCATGCGTATT	GATCACACATCTCGGCAACA
	cul1a	CAAGAACCCAGAGGAGGCAG	AGCCACACGCTTGCTTTAGT
	cul1b	TCGTGTACCTGTCATCAAGAAAT	GGTATGTGTGCGTCCACTCT
	cul2	TGTTCGTGGAGTCGGTGTTAG	TTCTCCGTCATTCCTTTAGCC
	cul3	GAAGGACACCAAGATGCGGA	AGCACGTCTTCTCGAACTTTG
	cdc25b	CTCGCTGTTCTTGGAGGTCAA	CCACACTCCATAACGGGAGG
	myca	TCTGACGCCACTTATGCTGC	ACTCACCGGCATTTTGACACTTG
	mycb	TTTACCACGGCTACGGCACT	ACCACATCGTCCCCCAAAAA
	dhfr	CAGAGAACTCAAGACAGCCC	TGAGGATGCGGGTTACAAACA
	e2f1	CGCTTCAAATGGACCCACAC	GTCGATACGATTCCCTAGCCA
	e2f3	GTGCCTGACCCAAAGGAGAGTC	CCGTTGACGCCACCGTTT
	foxa1	ATAATGATGCACAAGAGGTCTATTC	GCTGGGTTCGCATAGGACA
	rangap1a	GGCAACAGTCTGGGTGAAGA	GAGGCGTGGAGAACTGGTTTA
	tk1	CCACGGAAGACAAGAGGACAG	CAACGGTGTCTGGGAAAAACTG
	rb1	AGCAGACGGAAGCAAATCCA	CAACGCAGGGAAAGCATCTG
	p53	GGGCAATCAGCGAGCAAA	ACTGACCTTCCTGAGTCTCCA
	puma	TGGAAAGCAGAGTGGACGAA	GATGGCAGGGCTGGATGA
	baxa	CGTTGTTCGTATTGGCAGTGG	TGGCTGGGGTCACTTTTCTC
	bcl2a	CGAGTTTGGTGGGACCATGT	CGTACATCTCCACGAAGGCA
	mdm2	AAGCAGTGATCCTGAGAGTTC	ATCCGAAGACTCGCTGTTC
	mdm4	ATTCGGATGCGGCAAAGA	CCCACTGGACACCTGACC
	caspase8	GGCACTGATATGGACAAAGATAGA	GCATCCGGCAAAAGGCAAAG
	caspase10	TTGAGAATTCGATGGTGGAGCA	ACTGTCGTGGTCGATGTCTG
